# Immunotherapy of triple-negative breast cancer with cathepsin D-targeting antibodies

**DOI:** 10.1186/s40425-019-0498-z

**Published:** 2019-02-04

**Authors:** Yahya Ashraf, Hanane Mansouri, Valérie Laurent-Matha, Lindsay B. Alcaraz, Pascal Roger, Séverine Guiu, Danielle Derocq, Gautier Robin, Henri-Alexandre Michaud, Helène Delpech, Marta Jarlier, Martine Pugnière, Bruno Robert, Anthony Puel, Lucie Martin, Flavie Landomiel, Thomas Bourquard, Oussama Achour, Ingrid Fruitier-Arnaudin, Alexandre Pichard, Emmanuel Deshayes, Andrei Turtoi, Anne Poupon, Joëlle Simony-Lafontaine, Florence Boissière-Michot, Nelly Pirot, Florence Bernex, William Jacot, Stanislas du Manoir, Charles Theillet, Jean-Pierre Pouget, Isabelle Navarro-Teulon, Nathalie Bonnefoy, André Pèlegrin, Thierry Chardès, Pierre Martineau, Emmanuelle Liaudet-Coopman

**Affiliations:** 1 IRCM, INSERM, U1194 Univ Montpellier, ICM, 208, rue des Apothicaires, F-34298 Montpellier, Cedex 5, France; 2 Department of Pathology, CHU Nîmes, Nîmes, France; 3 Department of Medical Oncology, ICM, Montpellier, France; 4 Biometry Department, ICM, Montpellier, France; 5 BIOS group, INRA, CNRS, Nouzilly, France; 6 Univ La Rochelle, UMR CNRS, 7266 La Rochelle, France; 7 Translational Research Unit, ICM, Montpellier, France; 8 Réseau d’Histologie Expérimentale de Montpellier, BioCampus, UMS3426 CNRS-US009 INSERM-UM, Montpellier, France

**Keywords:** TNBC, Human antibody-based therapy, Immunomodulation, Protease, Tumor microenvironment, Phage display

## Abstract

**Background:**

Triple-negative breast cancer (TNBC) treatment is currently restricted to chemotherapy. Hence, tumor-specific molecular targets and/or alternative therapeutic strategies for TNBC are urgently needed. Immunotherapy is emerging as an exciting treatment option for TNBC patients. The aspartic protease cathepsin D (cath-D), a marker of poor prognosis in breast cancer (BC), is overproduced and hypersecreted by human BC cells. This study explores whether cath-D is a tumor cell-associated extracellular biomarker and a potent target for antibody-based therapy in TNBC.

**Methods:**

Cath-D prognostic value and localization was evaluated by transcriptomics, proteomics and immunohistochemistry in TNBC. First-in-class anti-cath-D human scFv fragments binding to both human and mouse cath-D were generated using phage display and cloned in the human IgG1 λ format (F1 and E2). Anti-cath-D antibody biodistribution, antitumor efficacy and in vivo underlying mechanisms were investigated in TNBC MDA-MB-231 tumor xenografts in nude mice. Antitumor effect was further assessed in TNBC patient-derived xenografts (PDXs).

**Results:**

High *CTSD* mRNA levels correlated with shorter recurrence-free survival in TNBC, and extracellular cath-D was detected in the tumor microenvironment, but not in matched normal breast stroma. Anti-cath-D F1 and E2 antibodies accumulated in TNBC MDA-MB-231 tumor xenografts, inhibited tumor growth and improved mice survival without apparent toxicity. The Fc function of F1, the best antibody candidate, was essential for maximal tumor inhibition in the MDA-MB-231 model. Mechanistically, F1 antitumor response was triggered through natural killer cell activation via IL-15 upregulation, associated with granzyme B and perforin production, and the release of antitumor IFNγ cytokine. The F1 antibody also prevented the tumor recruitment of immunosuppressive tumor-associated macrophages M2 and myeloid-derived suppressor cells, a specific effect associated with a less immunosuppressive tumor microenvironment highlighted by TGFβ decrease. Finally, the antibody F1 inhibited tumor growth of two TNBC PDXs, isolated from patients resistant or not to neo-adjuvant chemotherapy.

**Conclusion:**

Cath-D is a tumor-specific extracellular target in TNBC suitable for antibody-based therapy. Immunomodulatory antibody-based strategy against cath-D is a promising immunotherapy to treat patients with TNBC.

**Electronic supplementary material:**

The online version of this article (10.1186/s40425-019-0498-z) contains supplementary material, which is available to authorized users.

## Background

Breast cancer (BC) is one of the leading causes of death in women in developed countries. Triple-negative breast cancer (TNBC), defined by the absence of estrogen receptor (ER), progesterone receptor (PR) and human epidermal growth factor receptor 2 (HER-2) overexpression and/or amplification, accounts for 15–20% of all BC cases [1]. Chemotherapy is the primary systemic treatment, but resistance to this treatment is common [1]. Hence, tumor-specific molecular targets and/or alternative therapeutic strategies for TNBC are urgently needed. With the discovery of antigens specifically expressed in TNBC cells and the developing technology of monoclonal antibodies, immunotherapy is emerging as a novel promising option for TNBC [2].

Human cathepsin D (cath-D) is a ubiquitous, lysosomal, aspartic endoproteinase that is proteolytically active at low pH. Cath-D is overproduced and abundantly secreted by human epithelial BC cells [3] with expression levels in BC correlating with poor prognosis [4, 5]. Cath-D affects both cancer and stromal cells in the breast tumor microenvironment by increasing BC cell proliferation [3, 6, 7], fibroblast outgrowth [8, 9], tumor angiogenesis [10, 11], tumor growth and metastasis [6]. Human cath-D is synthesized as a 52-kDa precursor that is converted into an active 48-kDa single-chain intermediate in the endosomes, and then into a fully active mature form, composed of a 34-kDa heavy chain and a 14-kDa light chain, in the lysosomes. Its catalytic site includes two critical aspartic residues, residue 33 on the 14-kDa chain and residue 231 on the 34-kDa chain.

The over-production of cath-D by BC cells leads to hypersecretion of the 52-kDa pro-cath-D into the extracellular environment [3]. Purified 52-kDa pro-cath-D auto-activates in acidic conditions giving rise to a catalytically active 51-kDa pseudo-cath-D that retains the 18 residues [27–44] of the pro-segment [12]. Extracellular cath-D displays oncogenic activities by proteolysis at acidic pH and via non-proteolytic mechanisms through protein-protein interaction [13–15]. Extracellular cath-D can modify the local extracellular matrix by cleaving chemokines [16, 17], growth factors, collagens, fibronectin, proteoglycans, protease inhibitors (e.g.*,* cystatin C [18], PAI 1 [19]), or by activating enzyme precursors (e.g.*,* cathepsins B and L) [13]. It can also promote BC cell proliferation by binding to an unknown receptor via the residues 27–44 of its pro-peptide [20]. It can trigger breast fibroblast outgrowth upon binding to the LRP1 receptor [9, 21], and induce endothelial cell proliferation and migration via the ERK and AKT signaling pathways [11]. Therefore, extracellular cath-D could represent a novel molecular target in BC.

Cath-D deficiency in humans is associated with neuronal ceroid lipofuscinosis, one amongst the most common pediatric neurodegenerative lysosomal storage diseases, indicating its non-redundant essential role in protein catabolism and cellular homeostasis maintenance [22]. Consequently concomitant inhibition of intracellular and extracellular cath-D with cell-permeable chemical drugs [23] could be toxic. Therefore, we hypothesized that human antibodies targeting extracellular cath-D, which is massively secreted in BC, could circumvent this toxicity and be better therapeutic agents.

Here, we first validated the potential value of cath-D as a tumor-specific extracellular target in TNBC and its suitability for antibody-based therapy. Then, we generated two human anti-cath-D scFv fragments cloned in the human IgG1 λ format (F1 and E2) that efficiently bind to human and mouse cath-D, even at the acidic pH of the TNBC microenvironment. F1 and E2 accumulated in TNBC MDA-MB-231 tumor xenografts, inhibited tumor growth and improved mice survival without apparent toxicity. Using this xenograft model, we found that the Fc function of F1 was essential for maximal tumor inhibition. Mechanistically, F1-based therapy triggered an antitumor response via natural killer (NK) cell activation, and antitumor cytokine production. Furthermore, F1 treatment prevented the recruitment of tumor-associated macrophages (TAMs) and myeloid-derived suppressor cells (MDSCs) within the tumor, a specific effect associated with a less immunosuppressive tumor microenvironment. Finally, F1 inhibited tumor growth of TNBC patient-derived xenografts (PDXs). This preclinical proof-of-concept study validates the feasibility and efficacy of an anti-cath-D immunomodulatory antibody-based strategy to treat patients with TNBC.

## Methods

### Reagents

The anti-human cath-D antibody against 52-, 48-, and 34-kDa forms was from Transduction Laboratories (#610801 BD). The anti-human cath-D antibody (ab75811) against 14-kDa form was from Abcam. The anti-human cath-D antibody against 4-kDa pro-domain was kindly provided by Prof M. Fusek (Oklahoma Medical Research Foundation). The anti-human cath-D antibodies M1G8, D7E3 and M2E8 were previously described [9, 24]. The anti-human cath-D antibody (clone C-5) and CD11c (HL3) were from Santa Cruz Biotechnology. The anti-human Fc antibody conjugated to HRP (A0170) was from Sigma Aldrich. The anti-human CD20 chimeric IgG1 antibody (rituximab) was from Roche, and the anti-mouse F4/80 antibody (clone BM8, MF48000) from Invitrogen. Matrigel (10 mg/ml) was purchased from Corning. The fluorescent-conjugated antibodies against CD45 (30-F11), F4/80 (BM8), CD11b (M1/70), and Gr1 (RB6-8C5) were from Thermo Fisher Scientific, and against CD49b (DX5), and MHC-II (M5/114.15.2) were from Abcam. Recombinant human pro-cath-D was purchased from R&D Systems.

### Cell lines, ELISA, immunoprecipitation and western blotting

The MDA-MB-231 cell line was previously described [6]. Cells were cultured in DMEM with 10% fetal calf serum (FCS, GibcoBRL). To produce conditioned medium, cells were grown to 90% confluence in DMEM medium with 10% FCS, and conditioned medium was centrifuged at 800 x g for 10 min. For sandwich ELISA, 96-well plates were coated with M2E8 antibody in PBS (500 ng/well) at 4 °C overnight. After blocking non-specific sites with PBST/1% BSA, conditioned medium was added at 4 °C for 2 h. After washes in PBST, serial dilutions of F1 or E2 were added at 4 °C for 2 h and interaction revealed with an anti-human Fc antibody conjugated to HRP (1/2000; 355 ng/well). Cath-D was quantified in TNBC cytosols by sandwich ELISA, as described above, after coating with the D7E3 antibody in PBS (200 ng/well) and with the M1G8 antibody conjugated to HRP (1/80), and using recombinant pro-cath-D (1.25-15 ng/ml) for reference [6]. TNBC cytosols were previously prepared and frozen [25]. GST-cath-D fusion proteins were produced in the *E. coli* B strain BL21 as described [9]. The resulting proteins were separated on 12% SDS-PAGE and analyzed by immunoblotting.

### Study approval

Mouse experiments were performed in compliance with the French regulations and ethical guidelines for experimental animal studies in an accredited establishment (Agreement No. D3417227). The study approval for PDXs was previously published [26]. For TMA, TNBC samples were provided by the biological resource center (Biobank number BB-0033-00059) after approval by the Montpellier Cancer Institute Institutional Review Board, following the Ethics and Legal national French dispositions for the patients’ information and consent. For BC cytosols, patient samples were processed according to the French Public Health Code (law n°2004–800, articles L. 1243–4 and R. 1243–61), and the biological resources center has been authorized (authorization number: AC-2008-700; Val d’Aurelle, ICM, Montpellier) to deliver human samples for scientific research. All patients were informed before surgery that their surgical specimens might be used for research purposes.

### In vivo studies

MDA-MB-231 cells (2× 10^6^; mixed 1:1 with Matrigel) were injected subcutaneously in 6-week-old female athymic mice (Foxn1^nu^, ENVIGO). When tumors reached a volume of about 50 mm^3^, tumor-bearing mice were randomized and treated with F1 (15 mg/kg), E2 (15 mg/kg), rituximab (15 mg/kg), or NaCl by intraperitoneal injection 3 times per week. Tumors were measured using a caliper and volume was calculated using the formula *V* = (tumor length × tumor width × tumor depth)/2, until the tumor volume reached 2000 mm^3^. For PDX models, approximately 5 × 5 × 5 mm of B1995 and B3977 tumor fragments were transplanted in the inter-scapular fat pads of in 6-week-old female Foxn1^nu^ mice. When tumor volume reached a volume of about 150 mm^3^, mice were randomized in two treatments groups: F1 (15 mg/kg) or saline solution by intraperitoneal injection 3 times per week. Tumor volumes were measured as described above.

### SPECT/CT imaging

To generate ^177^Lu-labeled antibodies, F1 and E2 were conjugated with p-SCN-benzyl-DOTA. The immunoreactivity of the DOTA-conjugated antibodies (5 and 7 DOTA/IgG for F1 and E2, respectively) was verified by ELISA. DOTA-conjugated F1 and E2 were then labeled with ^177^Lu (Perkin Elmer) at 200 MBq/mg. Radiochemical purity was > 97% and radionuclide purity > 99.94%. For SPECT-CT imaging, 5 mice were xenografted with MDA-MB-231 cells. When tumors reached a volume of about 150 mm^3^, mice received an intraperitoneal injection of 7 MBq of ^177^Lu-F1 or ^177^Lu-E2 (3 mice for F1 and 2 for E2). At 24, 48, and 72 h post-injection, whole-body SPECT/CT images were acquired using a four-headed NanoSPECT imager (Bioscan Inc., Washington DC). Reconstructed data from SPECT and CT images were visualized and co-registered using Invivoscope®.

### Immunohistochemistry

For cath-D immunostaining, TNBC TMA and PDX primary tumor sections were incubated with anti-cath-D mouse antibody (clone C-5) at 0.4 μg/ml for 20 min after heat-induced antigen retrieval using the PTLink pre-treatment (Dako) and the High pH Buffer (Dako) and endogenous peroxidase quenching with Flex Peroxidase Block (Dako). After two rinses in EnVision™ Flex Wash buffer (Dako), sections were incubated with a HRP-labeled polymer coupled to secondary anti-mouse antibody (Flex® system, Dako) for 20 min, followed by 3,3′-diaminobenzidine as chromogen. Sections were counterstained with Flex Hematoxylin (Dako) and mounted after dehydration. Sections were analyzed independently by two experienced pathologists, both blinded to the tumor characteristics and patient outcomes at the time of scoring. In tumor and normal epithelial breast cells with peripheral membrane labeling, cath-D signal was scored as absent (0%), low (< 20%), moderate (20–50%), or high (> 50%). Extracellular granulations observed in the stroma were considered as extracellular cath-D staining. Extracellular stromal cath-D signal was defined as: absent (0%), low (< 20%), moderate (20–50%), or (high > 50%). For IHC of MDA-MB-231 xenografts, tumor samples were collected and fixed in 10% neutral buffered formalin for 24 h, dehydrated, and embedded in paraffin. For F4/80 immunostaining, xenograft sections (4-μm thick) were incubated with an anti-F4/80 antibody for 30 min, followed by a rabbit anti-rat antibody (Thermo Scientific, 31,218) before the Envision® system (Dako) as described above. F4/80 staining images were digitalized with the NanoZoomer slide scanner (Hamamatsu) and analyzed with the Aperio Imagescope software.

### Homology modeling and docking

Homology models were built using Modeller [27]. The heavy and light chain (VH and VL) were modeled separately, using as template the closest homolog with the same CDR length. VH and VL models were then reassembled based on the relative orientation in the template used for VH modeling. Docking of each molecular model on cath-D was made using PRIOR [28]. Figures were prepared using the PyMOL Molecular Graphics System (Version 2.0 Schrödinger, LLC).

### Gene expression data analysis

Recurrence-free survival with a 10-year follow-up was calculated using the on-line Kmplot tool accessed on October 22, 2017 with the 200766_at Affymetrix probe ( [29], www. http://kmplot.com). Analysis was restricted to the 255 patients with TNBC present in the database at this date and with the best cut-off option. The cut-off value was 1919 with a probe range from 50 to 6518. The group with high cath-D mRNA expression at the time 0 represented 38% of the population. Differences were evaluated with the Log-rank test.

### Quantitative RT-PCR

Reverse transcription of total RNA was performed at 37 °C using the Moloney murine leukemia virus reverse transcriptase (Invitrogen, Carlsbad, CA) and random hexanucleotide primers (Promega, Madison, WI). Real-time quantitative PCR analyses were performed on a Light Cycler 480 SYBR Green I master and a Light Cycler 480 apparatus (both from Roche Diagnostics, Indianapolis, IN). The PCR product integrity was verified by melting curve analysis. Quantification data were normalized to the amplification data for the reference gene encoding ribosomal protein S9 (*RPS9*). The sequences of the primers for *IL-15*, *GZMB*, *PRF1*, *IFNγ, CD206*, *F4/80*, *TGFβ,* and *RPS9* are presented  (Additional file 1: Table S1).

### Isolation of tumor-infiltrating cells and FACs analysis

Tumors were digested with a mixture of collagenase IV (1 mg/ml) (Sigma) and DNase I (200 U/ml) (Sigma) in Hank’s Balanced Salt Solution (HBSS) containing 2% FCS at 37 °C for three incubations of 15 min/each. The mixtures were then mechanically separated using the Gentle MACs procedure. After digestion, tumor suspensions were passed through a 70 μm nylon cell strainer, centrifuged and resuspended in FACS buffer (PBS pH 7.2, 1% FBS, 2 mM EDTA and 0.02% sodium azide). Cells were blocked with FACS buffer containing 1% (*v*/v) of Fc Block (Miltenyi) and, stained with fluorescent-conjugated antibodies against the following cell surface markers: CD45, CD49b, F4/80, CD11c, CD11b, Gr1 and MHC-II. MDSCs were defined as CD45^pos^CD49b^neg^CD11c^neg^CD11b^pos^Gr1^pos^MHC-II^neg^ cells. Dendritic cells were defined as CD45^pos^CD49b^neg^CD11c^pos^MHC-II^neg^ cells. Macrophages were defined as CD45^pos^CD11b^pos^F4/80^pos^ cells within the gate excluding MDSCs and dendritic cells. Sorted cells were then washed in FACS buffer, and fixed with 1% PFA in PBS. Samples were analyzed by flow cytometry using a Beckman and Coulter Cytoflex flow cytometer. Tumor cells were defined as CD45-negative events in a scatter gate that included small and large cells. Events were analyzed with FlowJo 10.4.

### Statistical analysis

A linear mixed regression model was used to determine the relationship between tumor growth and number of days after xenograft. The variables included in the fixed part of the model were the number of days post-graft and the treatment group; their interaction was also evaluated. Random intercepts and random slopes were included to take into account the time effect. The model coefficients were estimated by maximum likelihood. A survival analysis was conducted, and the event considered was a tumor volume of 2000 mm^3^. Survival rates were estimated using the Kaplan-Meier method and survival curves were compared with the Log-rank test. Statistical analysis was conducted with the STATA 13.0 software. The Student’s *t* test was used to evaluate difference. Statistical significance was set at the 0.05 level.

## Results

### Cath-D within the tumor microenvironment is eligible for antibody-mediated targeted therapy in TNBC patients

**Fig. 1:**
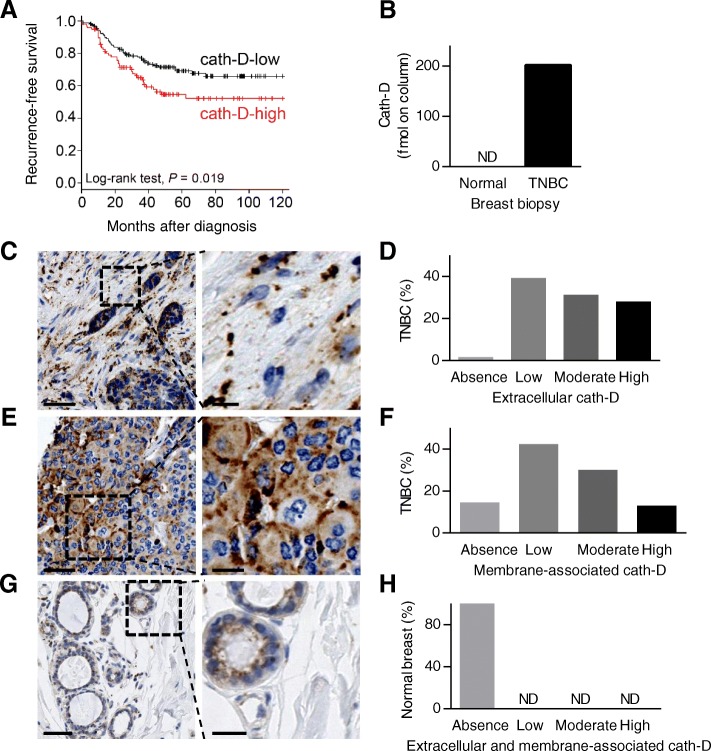
Cath-D is eligible for antibody-mediated targeted therapy in TNBC. **a** Kaplan-Meier curves of recurrence-free survival according *CTSD* mRNA expression in TNBC. *n* = 255 patients with TNBC; HR = 1.65 [1.08–2.53], *P* = 0.019, log-rank test. **b** Detection of extracellular and cell-surface cath-D in TNBC by proteomics. A TNBC biopsy and its paired normal breast tissue were biotinylated followed by protein digestion. The corresponding glycoprotein fractions containing proteins that are located either in the extracellular matrix or at the exterior face of the plasma membrane were analyzed by proteomics. **c** Detection of extracellular cath-D in a TNBC TMA. Cath-D was monitored in a TMA by IHC using a monoclonal anti-human cath-D (C-5; sc-377,127) antibody. Scale bar, 50 μm (left panel). Higher magnifications of the boxed regions showing extracellular cath-D (right panel). Scale bar, 10 μm. **d** Quantification of extracellular cath-D in a TNBC TMA. *n* = 123 samples. **e** Detection of membrane-associated cath-D at the cancer cell surface in a TNBC TMA. Scale bar, 50 μm (left panel). Higher magnifications of the boxed regions showing perimembranous cath-D (right panel). Scale bar, 20 μm. **f** Quantification of membrane-associated cath-D at the cancer cell surface in a TNBC TMA. *n* = 123 samples. **g** Detection of cath-D in normal breast tissue. Scale bar, 50 μm (left panel). Higher magnifications of the boxed regions (right panel). Scale bar, 20 μm. **h** Quantification of extracellular and membrane-associated cath-D in a normal breast TMA. *n* = 50 samples

First, we investigated the clinical significance of the expression of *CTSD* (the gene encoding cath-D) in a cohort of 255 patients with TNBC using an online survival analysis [29]. High *CTSD* mRNA level was significantly associated with shorter recurrence-free survival (HR = 1.65 [1.08–2.53]; *p* = 0.019) (Fig. 1a), suggesting that cath-D overexpression could be used as a predictive marker of poor TNBC prognosis. Then, we assessed cath-D expression in cytosols of primary BC samples (HR^+^/HER2^+^; HR^−^/HER2^+^; HR^+^/HER2^−^, HR^−^/HER^−^) by cytosolic assay. Our results revealed a mean cath-D level of 43.5 pmol/mg protein in the TNBC (HR^−^/HER^−^) subtype that was not significantly different from that of the other BC subtypes **(**Additional file 2: Figure S1**)**, and was in the range of the cut-off values reported in clinical studies on all combined BC subtypes [4, 5]. Next, to assess whether cath-D in TNBC was an accessible molecular target for anti-cath-D antibodies, we re-analyzed cath-D status in previously published datasets used for biotin-based affinity isolation and proteomic analysis of accessible protein biomarkers in human BC tissues [30] and (Additional file 3: Figure S2). We found that extracellular and/or membrane-associated cath-D could be detected only in the TNBC tumor sample and not in the adjacent normal breast tissue (Fig. 1b). We validated these proteomic data by anti-cath-D immunohistochemistry (IHC) analysis of a Tissue Micro-Array (TMA) that included 123 TNBC samples (Additional file 4: Figure S3). We detected extracellular cath-D in the microenvironment of 98% of TNBC samples (Fig. 1c and d), and membrane-associated cath-D at the cancer cell surface of 85.7% of samples (Fig. 1e and f). Conversely, extracellular and membrane-associated cath-D was not detectable in normal breast tissues (Fig. 1g and h). Together with the previously published data, our results show that cath-D is a tumor cell-associated extracellular biomarker and strongly suggest that it could be a good candidate for antibody-based therapy in TNBC.

### Selection of novel anti-human cath-D scFv fragments by phage display

For future potential clinical application especially in patients with TNBC, we decided to engineer a collection of novel, fully human, anti-cath-D antibodies. For this purpose, we probed the phage antibody expression library Husc I [31, 32] with recombinant human 34 + 14-kDa cath-D, and isolated polyclonal human antibodies in scFv format showing specific binding to immobilized cath-D by ELISA. After enrichment by four rounds of bio-panning (Additional file 5: Figure S4A), we selected five monoclonal antibodies based on their binding to recombinant human 52-kDa pro-cath-D and 34 + 14-kDa mature cath-D (Additional file 5: Figure S4B). We purified these five his-tagged scFv fragments by affinity chromatography (Additional file 5: Figure S4C), and determined by ELISA that the purified antibodies still bound to secreted human 52-kDa pro-cath-D and cellular cath-D (Additional file 5: Figure S4D) from MDA-MB-231 cells (cell line derived from an invasive ductal cell carcinoma that represents one of the most common TNBC models). These scFv fragments also recognized mouse cellular cath-D (81.1% of identity with human cath-D) from MEF cells (Additional file 5: Figure S4E). We then used the three scFv antibodies (F1, E2 and E12 scFv) with the highest binding to human and mouse cath-D to produce fully human IgG1 λ (F1, E2, E12).

### Generation of anti-cath-D human antibodies

**Fig. 2:**
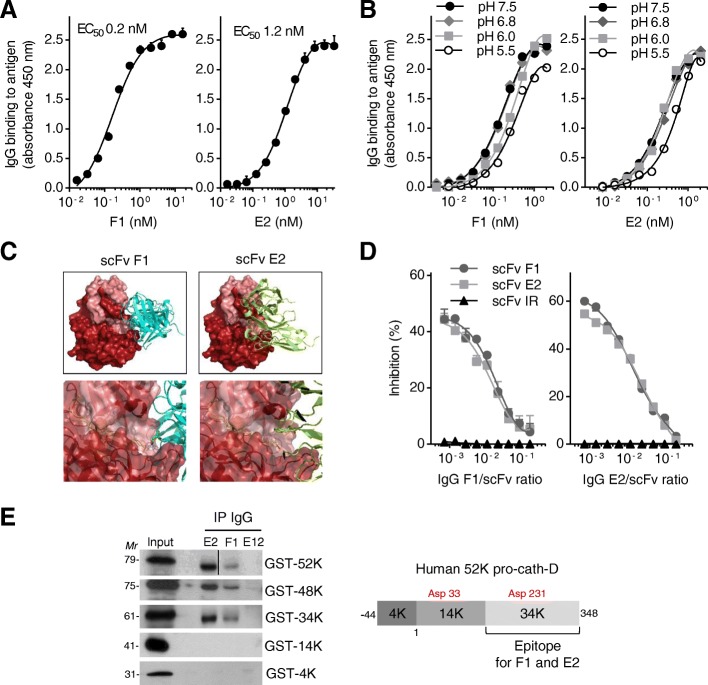
Characterization of the anti-cath-D F1 and E2 antibodies. **a** Binding of F1 and E2 to pro-cath-D secreted from MDA-MB-231 cells. Sandwich ELISA in which pro-cath-D from conditioned medium of MDA-MB-231 cells was added to wells pre-coated with anti-pro-cath-D M2E8 mouse monoclonal antibody in the presence of increasing concentrations of F1 (left panel) or E2 (right Panel). Binding of F1 and E2 to pro-cath-D was revealed with an anti-human Fc HRP-conjugated antibody. The EC_50_ values are shown. **b** Binding of F1 and E2 to pro-cath-D secreted from MDA-MB-231 cells at acidic pH. Sandwich ELISA was performed as described in (**a**) but at different pH values (7.5–5.5). **c** Molecular docking of the scFv F1 and E2. Ribbon representation of the scFv F1 (magenta) and scFv E2 (green) interface with the contact surface of mature cath-D (upper panels). Docking model in which the space-filled view of protruding L1 CDR inserts into cath-D catalytic site (bottom panels). **d** Competitive ELISA. Sandwich ELISA was performed as described in (**a**) with 1 nM F1 or E2 and increasing concentrations of scFv F1, E2 or IR (negative scFv). **e** Immunoprecipitation of GST-cath-D isoforms with F1 and E2. GST-cath-D isoforms were immunoprecipitated with F1, E2, or E12, and detected by immunoblotting using the relevant antibodies (left panels). *Mr*, relative molecular mass (kDa). Schematic representation of the human 52-kDa pro-cath-D sequence (right panel). The 4-kDa cath-D pro-fragment, 14-kDa light, and 34-kDa heavy mature chains are indicated. The intermediate 48-kDa form (not shown) corresponds to the non-cleaved 14 + 34 kDa chains. The catalytic aspartate 33 and 231 (red) are shown

Sandwich ELISA using pro-cath-D secreted from MDA-MB-231 cells showed that F1 (Fig. 2a, left panel) and E2 (Fig. 2a, right panel) retained good binding capacities (EC_50_ = 0.2 nM and 1.2 nM, respectively). Conversely, E12 in the IgG1 format lost its binding activity (data not shown). Moreover, F1 and E2 binding to pro-cath-D was comparable at pH values from 7.5 to 5.5 (Fig. 2b), suggesting that they are active also in the highly acidic tumor microenvironment. We also confirmed F1 and E2 good selectivity towards pro-cath-D compared with other aspartic enzymes, such as pro-cathepsin E, pepsinogen A and pepsinogen C (Additional file 6: Figure S5).

We next characterized the cath-D epitope recognized by F1 and E2. Molecular docking performed on the three-dimensional structure of mature cath-D (PDB ID 1LYA) [33] strongly suggested that F1 and E2 scFv interact mainly with the 34-kDa cath-D chain (in red) (Fig. 2c, top panels). Moreover, the third complementary determining region of the heavy chain (CDRH3) of both F1 and E2 scFv, which is crucial for antibody specificity, might protrude into the proteinase active site (Fig. 2c, bottom panels). By competitive ELISA, we confirmed that F1 and E2 epitopes overlapped (Fig. 2d). Finally, using GST-cath-D fusion fragments, we showed that both F1 and E2 immunoprecipitated the 52-, 48- and 34-kDa forms of GST-cath-D, but not the 4-kDa GST-cath-D pro-fragment and the 14-kDa light chain GST-cath-D (Fig. 2e, left panel). These results indicated that the cath-D epitopes of F1 and E2 are located mainly on the 34-kDa part of the protein (Fig. 2e, right panel).

### Anti-cath-D human antibodies localize and accumulate in MDA-MB-231 tumor xenografts

**Fig. 3:**
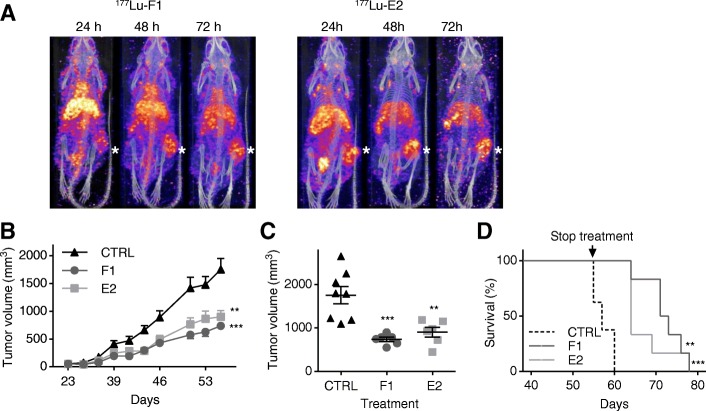
The anti-cath-D F1 and E2 antibodies accumulate in MDA-MB-231 tumor xenografts, reduce tumor growth in vivo and improve survival. **a** SPECT/CT analysis. MDA-MB-231 cells were xenografted subcutaneously in the right flank of nude mice. When tumor volume reached 150 mm^3^, mice received one intra-peritoneal injection of ^177^Lu- F1 or ^177^Lu- E2. Representative SPECT/CT images at 24, 48 and 72 h post-injection of ^177^Lu- F1 (left panels) and ^177^Lu- E2 (right panels). * shows tumors. **b** Tumor growth. MDA-MB-231 cells were subcutaneously injected in nude mice. When tumor volume reached 50 mm^3^, mice were treated with F1 (*n* = 6) or E2 (*n* = 6) (15 mg/kg), or NaCl (CTRL; *n* = 8) three times per week for 32 days. Mice were sacrificed when tumor volume reached 2000 mm^3^. Tumor volume (in mm^3^) is shown as the mean ± SEM. ***, *P* < 0.001 for F1; ** *P* = 0.002 for E2 (mixed-effects ML regression test). **c** Mean tumor volumes at day 55. ***, *P* = 0.0005 for F1; **, *P* = 0.0026 for E2 (*t* test); mean ± SEM. **d** Kaplan-Meier survival analysis. ***, *P* = 0.0005 for F1; **, *P* = 0.0016 for E2

We then assessed F1 and E2 localization by SPECT/CT and their bio-distribution in nude mice xenografted subcutaneously with MDA-MB-231 cells. When tumor cell xenografts reached about 150 mm^3^, mice received one single intraperitoneal injection of antibodies labeled with lutetium 177 (^177^Lu-F1 and ^177^Lu-E2), a radionuclide emitting gamma particles that can be used for imaging and bio-distribution purposes. Whole-body SPECT/CT images acquired 24, 48 and 72 h post-injection showed that ^177^Lu-F1 and ^177^Lu-E2 accumulated in the MDA-MB-231 tumor xenografts (asterisks in Fig. 3a).

The bio-distribution profiles confirmed that ^177^Lu-F1 and ^177^Lu-E2 gradually accumulated in the tumors from 24 h and up to 96 h (Additional file 7: Figure S6). The percentage (mean ± SD) of injected activity/g tissue detected in tumors (%IA/g) at 72 h was 8.2% ± 4.3% for F1 and 8.1% ± 2.2% for E2 (Additional file 7: Figure S6). Moreover, at 72 h, ^177^Lu-F1 and ^177^Lu-E2 were present also in blood (6.4 ± 2.1 and 11.3% ± 6.1%, respectively), and liver (10.5 ± 5.2 and 10.7% ± 4.7%, respectively). However, their concentration in blood and liver decreased rapidly due to physiological elimination. These results indicate that F1 and E2 localize and accumulate in TNBC MDA-MB-231 xenografts.

### The anti-cath-D F1 and E2 antibodies inhibit TNBC MDA-MB-231 tumor growth and improve survival

We used athymic Foxn1^nu^ nude mice xenografted subcutaneously with MDA-MB-231 cells to study the antitumor properties of the anti-cath-D antibodies F1 and E2. When MDA-MB-231 tumors reached 50 mm^3^, we treated mice with F1, E2 (15 mg/kg), or saline solution (control) by intraperitoneal injection 3 times per week for 32 days (day 23–55 post-graft), and sacrificed them when tumor volume reached 2000 mm^3^. Treatment with F1 or E2 significantly delayed tumor growth compared with control (Fig. 3b; *P* < 0.001 for F1, *P* = 0.002 for E2). At day 55, tumor volume was reduced by 58% in the F1 (*P* = 0.0005) and by 49% (*P* = 0.0026) in the E2 group compared with control (Fig. 3c). Moreover, the overall survival rate, reflected by a tumor volume inferior to 2000 mm^3^, was significantly longer in mice treated with F1 or E2 than in controls, with a median survival of 72 and 64 days for the F1 and E2 groups respectively, compared with 57 days for control animals (Fig. 3d; Kaplan-Meier survival analysis, *P* = 0.0005 for F1, *P* = 0.0016 for E2). These results show that anti-cath-D human antibodies as monotherapy delay very efficiently tumor growth in nude mice xenografted with MDA-MB-231 cells.

### Anti-cath-D antibody-based therapy prevents macrophage recruitment within MDA-MB-231 tumor xenografts

**Fig. 4:**
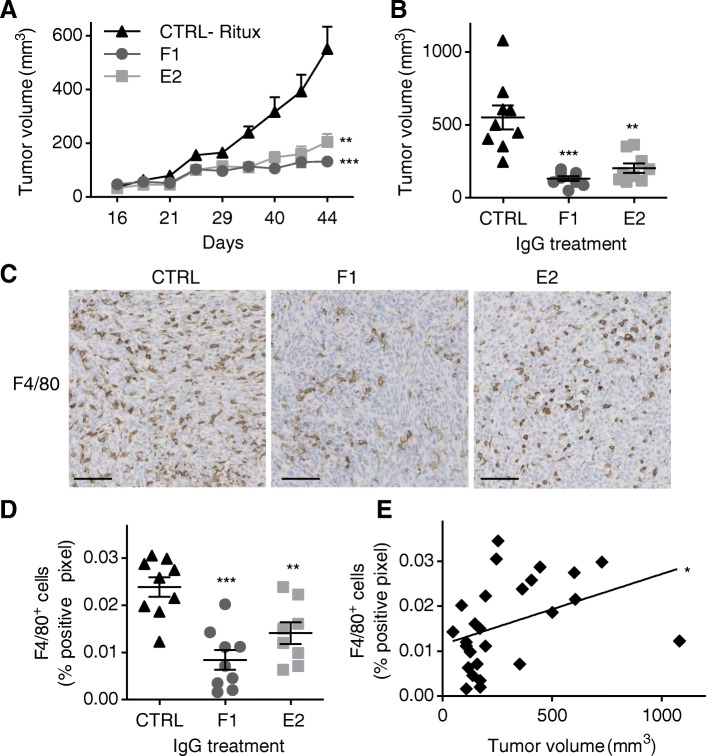
Anti-cath-D antibody-based therapy prevents macrophage recruitment within MDA-MB-231 tumor xenografts. **a** Tumor growth. When MDA-MB-231 tumor xenografts reached a volume of 50 mm^3^, nude mice were treated with F1 (*n* = 9), E2 (*n* = 9), or rituximab (CTRL; *n* =9) (15 mg/kg) for 28 days (day 16–44). At day 44, all mice were sacrificed. ***, *P* = 0.001 for F1; **, *P* = 0.002 for E2 (mixed-effects ML regression test). **b** Mean tumor volume at day 44. *n =* 9 for CTRL; *n =* 9 for F1; *n =* 9 for E2. ***, *P* = 0.0001 for F1; **, *P* = 0.0012 for E2 (Student’s *t* test); mean ± SEM. **c** Representative images of F4/80 immunostaining in MDA-MB-231 tumor cell xenografts from CTRL- (rituximab), F1- and E2-treated mice. Scale bars, 100 μm. **d** Quantification of F4/80^+^ macrophages. Percentage (mean ± SEM) of positive pixels relative to the total pixels; *n = *9 for rituximab (CTRL); *n = *9 for F1; *n =* 9 for E2; ***, *P* < 0.0001 for F1; **, *P* = 0.0063 for E2 (Student’s *t*-test). **e** Linear regression analysis of F4/80^+^ macrophages and tumor volumes. R^2^ = 0.1553; *, *P* = 0.0464, *n* = 27

To further investigate the in vivo mechanisms underlying the antitumor effect of F1 and E2, we treated nude mice xenografted with MDA-MB-231 cells with F1, E2 or the anti-human CD20 IgG1 rituximab, as negative isotype control (same schedule as before), and then sacrificed all mice at the treatment end. F1 and E2 led to a significant inhibition of tumor growth compared with rituximab (*P* = 0.001 for F1, *P* = 0.002 for E2) (Fig. 4a). At the end of the experiment (day 44), tumor volume was reduced by 76% in the F1 group (*P* = 0.0001) and by 63% (*P* = 0.0012) in the E2 group, compared with the rituximab group (Fig. 4b). Moreover, although F1 and E2 cross-react with mouse cath-D, mice treated with the anti-cath-D antibodies gained weight and displayed normal activities (Additional file 8: Figure S7), suggesting minimal off-target effects for these human antibodies.

Then, we investigated the effect of F1 and E2 monotherapy on tumor cell proliferation, apoptosis, and angiogenesis by IHC. Ki67, a marker of proliferating cells (Additional file 9: Figure S8A and B), activated caspase 3, a marker of apoptosis (Additional file 9: Figure S8C and D), and the angiogenesis marker CD31 (Additional file 9: Figure S8E and F) were similarly expressed in tumors from the three groups of mice. As antibody-based immunotherapy is often associated with immune modulation of the tumor microenvironment [34], we assessed the impact of anti-cath-D antibodies on tumor-infiltrating immune cells, particularly on myeloid cells that are present in Foxn1^nu^ nude mice. Staining with the anti-macrophage F4/80 antibody revealed that macrophage infiltration in the tumor core was reduced by 64.8% in the F1 and by 41% in the E2 group, compared with the rituximab group (Fig. 4c and d; *P* < 0.0001 for F1, *P* = 0.0063 for E2). Moreover, the percentage of F4/80^+^ cells was positively associated with tumor volume by linear regression analysis (Fig. 4e; *P* = 0.0464). Our findings show that anti-cath-D antibody treatment inhibits macrophage infiltration in MDA-MB-231 tumor xenografts, suggesting that this antibody-based therapy may impact the tumor immune microenvironment.

### The anti-cath-D antibody F1 prevents M2-like macrophages and MDSC recruitment, leading to a less immunosuppressive tumor microenvironment in MDA-MB-231 xenografts

As the immunomodulatory effect of antibody-based therapy could depend on Fc-mediated mechanisms [35], we engineered an aglycosylated Fc-silent version of the F1 antibody (F1Fc) in which the mutation N297A prevents binding to FcγRs [36].

**Fig. 5:**
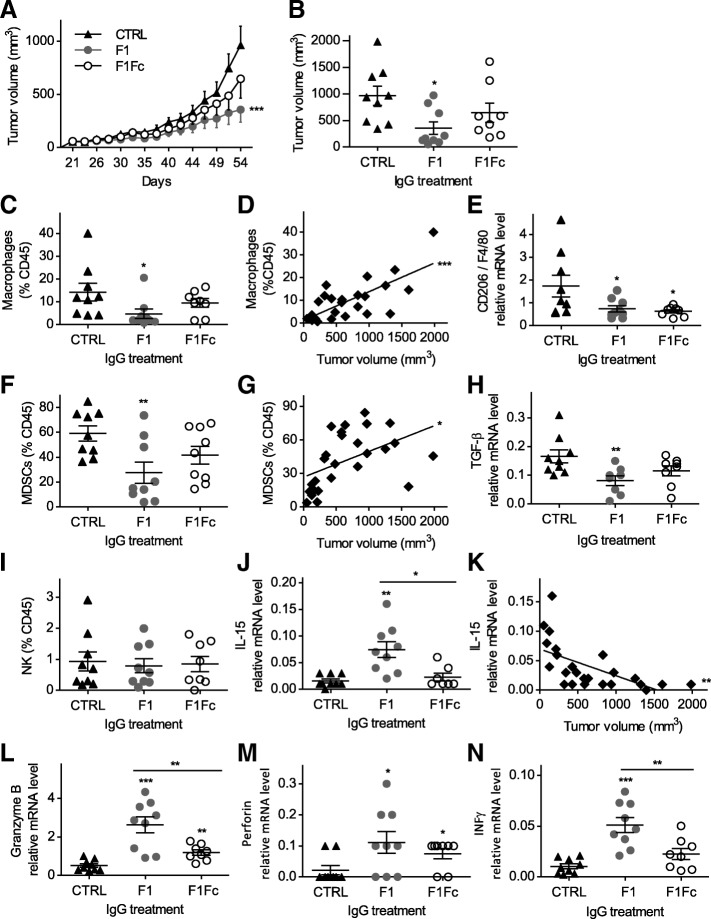
Anti-cath-D antibody-based therapy prevents M2-like macrophage and MDSC recruitment, and triggers antitumor response via NK cell activation in MDA-MB-231 xenografts. **a** Tumor growth. Nude mice bearing MDA-MB-231 tumors of 50 mm^3^ were treated with F1 (*n* = 9), F1Fc (*n* = 8), or rituximab (CTRL; *n* = 9) (15 mg/kg) for 35 days. At day 54, mice were sacrificed. *, *P* < 0.001 for F1 versus CTRL; *P* = 0.077 for F1Fc versus CTRL; *P* = 0.069 for F1 versus F1Fc (mixed-effects ML regression test). **b** Mean tumor volumes at day 54. Mean ± SEM; *, *P* = 0.011 for F1 versus CTRL; *P* = 0.231 for F1Fc versus CTRL, *P* = 0.189 for F1 versus F1Fc (Student’s *t*-test). **c** TAM recruitment. The percentage of F4/80^+^ CD11b^+^ TAMs was quantified by FACS and expressed relative to all CD45^+^ immune cells (*n* = 9 for CTRL; *n* = 9 for F1; *n* = 8 for F1Fc); *, *P* = 0.044 for F1 versus CTRL; *P* = 0.3 for F1Fc versus CTRL (Student’s *t*-test). **d** Linear regression analysis of TAM and tumor volumes. R^2^ = 0.5425; ***, *P* < 0.0001; *n* = 26. **e** Quantification of *CD206* mRNA expression. Total RNA was extracted from MDA-MB-231 tumor xenografts at the end of treatment, and *CD206* expression analyzed by RT-qPCR and shown relative to F4/80 (*n* = 9 for CTRL; *n* = 9 for F1; *n* = 8 for F1Fc); *P* = 0.05 for F1 versus CTRL; *P* = 0.04 for F1Fc versus CTRL (Student’s *t*-test). **f**  MDSC recruitment. The percentage of Gr1^+^ CD11b^+^ MDSCs was quantified by FACS analysis and expressed relative to all CD45^+^ cells (*n* = 9 for CTRL; *n* = 9 for F1; *n* = 8 for F1Fc); **, *P* = 0.008 for F1 versus CTRL; *P* = 0.079 for F1Fc versus CTRL (Student’s *t*-test). **g** Linear regression analysis of MDSC and tumor volumes. R^2^ = 0.23315; *, *P* = 0.0125; *n* = 26. **h** Quantification of *TGFβ* mRNA expression. Total RNA was extracted from MDA-MB-231 tumor cell xenografts at the end of treatment and *TGFβ* expression analyzed by RT-qPCR. Data are relative to *RPS9* expression (*n* = 9 for CTRL; *n* = 9 for F1; *n* = 8 for F1Fc); **, *P* = 0.009 for F1 versus CTRL; *P* = 0.1 for F1Fc versus CTRL (Student’s *t*-test). **i** NK recruitment. The percentage of CD49b^+^ CD11b^+^ NK cells was quantified by FACS and expressed relative to all CD45^+^ cells (mean ± SEM; *n* = 9 for rituximab (CTRL); *n* = 9 for F1; *n* = 8 for F1Fc); *P* = 0.7 for F1 versus CTRL; *P* = 0.8 for F1Fc versus CTRL; *P* = 0.8 for F1 versus F1Fc (Student’s *t*-test). **j** Quantification of *IL-15* mRNA expression. Total RNA was extracted from MDA-MB-231 tumor cell xenografts at the end of treatment and *IL-15* analyzed by RT-qPCR. Data are the mean ± SEM expression level relative to *RPS9* expression (*n* = 9 for rituximab (CTRL); *n* = 9 for F1; *n* = 8 for F1Fc); **, *P* = 0.0013 for F1 versus CTRL; *P* = 0.365 for F1Fc versus CTRL; *, *P* = 0.0127 for F1 versus F1Fc (Student’s *t*-test). **k** Linear regression analysis of *IL-15* mRNA level and tumor volumes. R^2^ = 0.3693; **, *P* = 0.0013; *n* = 26. **l** Quantification of *granzyme B* mRNA expression as in (**j**). ***, *P* = 0.0002 for F1 versus CTRL; **, *P* = 0.0011 for F1Fc versus CTRL; **, *P* = 0.0076 for F1 versus F1Fc (Student’s *t*-test). **m** Quantification of *perforin* mRNA expression as in (**j**). *, *P* = 0.033 for F1 versus CTRL; *, *P* = 0.0294 for F1Fc versus CTRL; *P* = 0.386 for F1 versus F1Fc (Student’s *t*-test). **n** Quantification of *IFNγ* mRNA expression as in (**j**). ***, *P* < 0.0001 for F1 versus CTRL; *P* = 0.0513 for F1Fc versus CTRL; **, *P* = 0.0078 for F1 versus F1Fc (Student’s *t*-test)

We first confirmed that F1Fc binding to cath-D was comparable to that of F1 (Additional file 10: Figure S9). We then treated mice harboring MDA-MB-231 tumor cell xenografts with F1Fc, F1 or rituximab (CTRL) as before. F1 treatment significantly reduced tumor growth compared with rituximab (Fig. 5a; *P* < 0.001). Conversely, F1Fc effect on tumor growth was reduced compared with F1 (Fig. 5a). At the end of the experiment (day 54), tumor volume was reduced by 63.1% (*P* = 0.01) in the F1 group and only by 32.9% (not significant) in the F1Fc group compared with the rituximab group (Fig. 5b). Thus, the Fc effector functions of F1 are essential for maximal tumor inhibition, corroborating the participation of immune cells in the antitumor response induced by anti-cath-D antibody therapy.

We next analyzed tumor immune infiltrates at day 54 by FACS analysis with a specific focus on TAMs and MDSCs, associated with tumor progression and relapse in BC [37, 38]. In agreement with the previous IHC results (Fig. 4c and d), the percentage of F4/80^+^ CD11b^+^ macrophages within the immune CD45^+^ cell population was significantly decreased by 67% in F1-treated animals (*P* = 0.044 compared with the rituximab group) and only by 33% in the F1Fc-treated group (not significant) (Fig. 5c). Moreover, linear regression analysis showed that the percentage of macrophages was significantly correlated with tumor volume in all animals (three treatment groups together) (R^2^ = 0.5425, *P* < 0.0001) (Fig. 5d), suggesting that in this model, tumor progression was associated with macrophage enrichment and that the F1 antibody prevented their infiltration. In many tumors including BC, TAMs are M2 polarized, which is associated with protumorigenic functions [37, 39]. At day 54, the expression of *CD206* mRNA, a M2-associated marker [40], was significantly downregulated by 56.6% (*P* = 0.05) and 62.9% (*P* = 0.04) in MDA-MB-231 tumor xenograft RNA samples from the F1- and F1Fc-treated group, respectively, compared with control (rituximab) (Fig. 5e). This suggests that anti-cath-D antibody monotherapy prevented tumor infiltration by M2 macrophages and that this could have contributed to limit tumor growth.

In addition, the percentage of Gr1^+^ CD11b^+^ MDSCs within the immune CD45^+^ cell population also was significantly decreased by 53.4% (*P* = 0.008) in F1-treated mice and by 29.6% in F1Fc-treated mice (not significant) compared with control (rituximab) (Fig. 5f). The percentage of tumor-infiltrating MDSCs was positively correlated with tumor volume in the whole population (three groups together) (Fig. 5g; *P* = 0.0125). Because of the changes of TAMs and MDSCs, F1 treatment may alter immunosuppressive factors in the tumor microenvironment. Indeed, mRNA expression of the inhibitory cytokine transforming growth factor β (*TGFβ*) was reduced by 51% in tumors from F1-treated mice (*P* = 0.0099 compared with the rituximab control) and by 30.6% in the F1Fc group (not significant) (Fig. 5h). This strengthened the effect of anti-cath-D antibody therapy on immunosuppressive M2 macrophages and MDSCs. Our data highlight the strong impact of anti-cath-D antibody therapy on the tumor immune microenvironment, leading to a less immunosuppressive microenvironment in MDA-MB-231 xenografts.

### The anti-cath-D antibody F1 antitumor response is triggered via NK cell activation

NK cells are needed for the efficacy of antibody-based immunotherapies by triggering antibody-dependent cell-mediated cytotoxicity [41]. To determine the potential implication of NK cells in anti-cath-D antibody therapy, we quantified by FACS analysis the CD49b^+^ CD11b^+^ NK cell population in tumors at the end of treatment (day 54) and found that it was comparable in the F1-, F1Fc- and rituximab-treated groups (Fig. 5i). RT-qPCR analysis of the expression of *IL-15*, a cytokine associated with NK cell activation [42], showed that it was upregulated (up to 209%, *P* = 0.0013 compared with rituximab) in the F1 group, but not in the F1Fc-treated group (*P* = 0.0127 compared with F1) (Fig. 5j). This suggests a causal relationship between the F1 antitumor response and NK cell activation. In agreement, *IL-15* mRNA level was inversely correlated with tumor volume in the entire population (three groups together) by linear regression analysis (Fig. 5k; *P* = 0.0013). We then quantified granzyme B (*GZMB*) and perforin 1 (*PRF1*) mRNA levels, as a read-out of NK cell activity. *GZMB* was strongly upregulated (up to 220%) in the F1 group (*P* = 0.0002 compared with rituximab). Although significant, the up-regulation remained modest in the F1Fc group compared with the control group and was significantly reduced compared with the F1-treated group (Fig. 5l; *P* = 0.0076). Similarly, *PRF1* expression was increased by 500% in the F1 group compared with control (*P* = 0.033) and slightly less in the F1Fc group (Fig. 5m). Finally, the mRNA expression of the antitumor cytokine *IFNγ* was upregulated by 494.8% in the F1 group compared with control (Fig. 5n; *P* < 0.0001). This upregulation was significantly reduced in the F1Fc-treated group compared with the F1 group (Fig. 5 n; *P* = 0.0078). Altogether, our results strongly suggest that the antitumor response of the anti-cath-D antibody F1 in MDA-MB-231 xenografts is in part triggered by Fc-dependent mechanisms via NK cell activation through IL-15 upregulation, associated with granzyme B and perforin production and the release of IFNγ.

### The anti-cath-D antibody F1 inhibits growth of patient-derived xenografts of TNBC

**Fig. 6:**
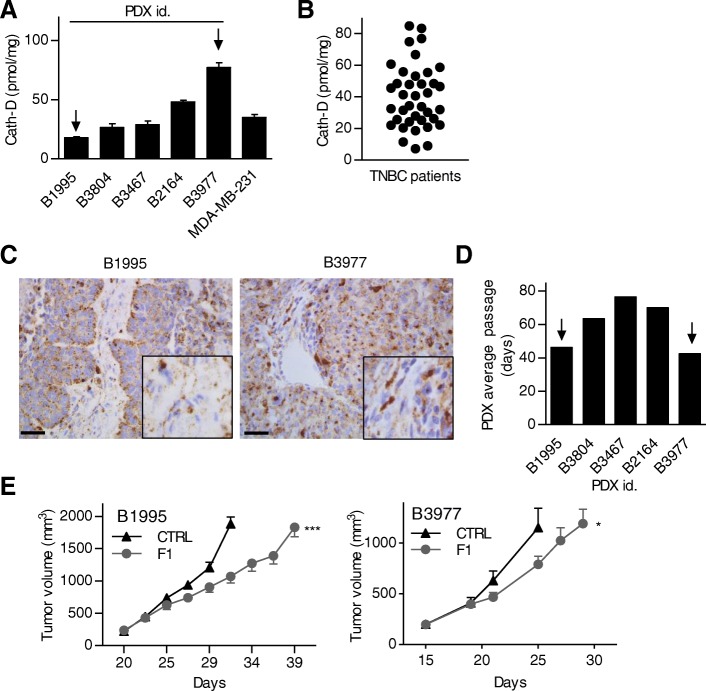
The anti-cath-D F1 antibody inhibits tumor growth of TNBC PDXs. **a** Cath-D expression in TNBC PDXs. Total cath-D expression was determined in whole cytosols from five TNBC PDXs by sandwich ELISA with immobilized anti-human cath-D D7E3 antibody and anti-human cath-D M1G8 antibody coupled to HRP. Total cath-D expression was quantified also in MDA-MB-231 tumor xenografts. **b** Cath-D expression in TNBC biopsies. Total cath-D expression was assessed in whole cytosols from TNBC biopsies as described in (**a**); *n* = 41. **c** Expression of cath-D in the PDX B1995 and B3977. Cath-D expression in the PDX B1995 (left panel) and PDX B3977 (right panel) primary tumors was monitored by IHC using a monoclonal anti-human cath-D (C-5; sc-377,127) antibody. Scale bar: 25 μm. Insets, high magnification of the boxed regions. **d** Average passage duration for the first three passages in the five TNBC PDXs. **e** Therapeutic effects of F1 in mice engrafted with PDX B1995 or PDX 3977. Mice were engrafted with PDX B1995 (left panel) or PDX B3977 (right panel) and when tumor volumes reached 150 mm^3^, mice were treated with F1 (15 mg/kg) or NaCl (CTRL) three times per week. Mice were sacrificed when tumor volume reached 2000 mm^3^ and the corresponding tumor growth curves were stopped. Tumor volume (in mm^3^) is shown as the mean ± SEM; For PDX B1995 : *n* = 7 for CTRL; *n* = 7 for F1. ***, *P* < 0.001 for F1. For PDX B3977 : *n* = 10 for CTRL; *n* = 10 for F1. *, *P* = 0.022 for F1

Finally, we tested F1 effect in mice harboring PDXs of TNBC [26]. First, quantification by sandwich ELISA in whole cytosolic extracts of five representative TNBC PDXs showed that cath-D concentration varied from 18 to 77 pmol/mg of total protein (Fig. 6a). These values were in the same range as those detected in whole cytosolic extracts prepared from MDA-MB-231 tumor xenografts (Fig. 6a), and from 40 TNBC samples (Fig. 6b). Immunostaining of the B1995 and B3977 primary tumors with an anti-cath-D antibody confirmed that cath-D was detected in tumor cells and microenvironment (Fig. 6c), as previously observed with the TNBC TMA (Fig. 1c and e). These results indicate these PDX models are representative of the disease, at least concerning cath-D expression. We then engrafted athymic nude mice with PDX B1995 or PDX B3977, the two PDXs showing the fastest growth in nude mice (average passage duration for the first three passages: 46 days for B1995 and 42 days for B3977) (Fig. 6d). Tumor volume increase was significantly slowed down in mice treated with F1 compared with control (Fig. 6e).

## Discussion

Here we described the discovery, characterization, and preclinical development of F1, a fully human anti-cath-D IgG1 antibody isolated from a human scFv phage display library. Our study indicates the anti-cath-D human antibody F1 is a candidate drug that could be translated into the clinic for the treatment of patients with TNBC. Our findings demonstrated F1 therapeutic efficacy in three different TNBC models (MDA-MB-231 cell xenografts and two TNBC PDXs). Moreover, they also confirmed that tumor-specific extracellular non-receptor oncoproteins are reliable molecular targets for antibody-based cancer therapy. Indeed, although monoclonal antibodies have a remarkable efficacy as anti-cancer therapeutics [34], only a limited number of soluble targets have been explored so far, and antibody therapy is mainly focused on receptor antigens on cancer cells as “druggable” targets. In BC, the most relevant example is trastuzumab (Herceptin®) to treat HER2^+^ tumors.

Our data show that cath-D is a pertinent target for antibody-based therapy in TNBC. A recent study reported that cath-D is overexpressed in 71.5% of the 504 TNBC analyzed and proposed a prognostic model for TNBC outcome based on node status, cath-D expression and Ki67 index [43]. Here, we found that high *CTSD* mRNA expression was significantly associated with shorter recurrence-free survival in a cohort of 255 TNBC samples [29]. Our re-analysis of previously published proteomic data [30] allowed describing the extracellular and cell-surface expression of cath-D in TNBC. Moreover, in the TNBC TMA, we detected extracellular cath-D in the tumor microenvironment in 98.4% of samples and at the surface of cancer cells in 85.4% of samples. Conversely, in normal breast, we observed cath-D expression only inside luminal breast cells. Altogether, our data strongly suggest that extracellular cath-D could be considered as a therapeutic target and a biomarker in TNBC.

To engineer anti-cath-D antibodies, we used the antibody phage display technology, an efficient methodology for the isolation of human monoclonal antibodies for therapeutic applications [34]. Importantly, the human scFv antibody format is suitable for incorporation of the binding specificity into therapeutic proteins and can be reformatted into intact IgG1, as shown here. Anti-human cath-D mouse monoclonal antibodies have been produced also using the hybridoma strategy [24]. However, their therapeutic effect was not investigated. Vetvicka’s group also developed antibodies against the amino acid stretch (aa 27–44) within the human cath-D pro-peptide. These antibodies inhibit human BC cell xenograft growth in nude mice [44].

Here, we validated the concept of targeting extracellular cath-D in the TNBC microenvironment using anti-cath-D human antibodies in the IgG1 format that allows future clinical applications. In vivo*,* F1 and E2 inhibited tumor growth and improved the overall survival without apparent toxicity. Moreover, F1, our most potent antibody, inhibited growth also of two TNBC PDXs (B1995, and B3977). PDX B1995 was isolated from a patient with TNBC after neo-adjuvant therapy, whereas PDX B3977 was isolated from a patient with TNBC resistant to neo-adjuvant chemotherapy. Thus, our findings strongly suggest that F1 may represent a new therapeutic strategy to treat patient with TNBC resistant to chemotherapy.

Monoclonal antibodies mediate tumor cell inhibition through multiple mechanisms of action, including direct target inhibition through antigen-binding (Fab) fragment engagement and immune cell modulation through their constant region Fc. Using the F1Fc mutant we showed that F1 Fc function is essential for maximal tumor growth inhibition in athymic nude mice xenografted with TNBC MDA-MB-231 cells. The IgG1 Fc portion can recruit and activate components of the complement system and innate immune effector cells, resulting in destruction of antibody-targeted cancer cells through complement-dependent cytotoxicity, antibody-dependent cellular phagocytosis, or antibody-dependent cellular cytotoxicity (ADCC) [34]. Evidence from multiple in vivo models suggests that antibody binding to cellular FcγRs is the major pathway for ADCC, which is classically mediated by NK cells [45]. Here, we demonstrated that F1 leads to NK cell activation via IL-15 upregulation, associated with increased granzyme B and perforin production to eliminate tumor cells, as well as higher levels of the antitumor IFNγ cytokine. Conspicuously, these effects were limited when using the aglycosylated F1Fc antibody in which the mutation N297A prevents binding to FcγRs. Aglycosylated antibodies retain antigen binding, as observed for F1Fc, but show defects in binding to FcγRs, and ADCC induction [36]. Altogether these results indicate that NK cells are implicated in F1 antitumor response, suggesting ADCC occurrence in vivo. ADCC is one of the immunotherapeutic strategies currently under investigation to awake the innate anti-cancer response. Recent studies and clinical trials have highlighted that manipulation of NK cell activation can be used as an immunotherapy in cancer, particularly against tumor metastases [41]. As Fc dependence of antibodies is a sine qua non requirement for their use in the clinic to ensure effectiveness, NK cell activation following F1 antibody treatment is a key finding.

We also found that F1 has a remarkable effect on the tumor immune microenvironment. First, it prevented the infiltration of M2-like macrophages in MDA-MB-231 tumors. Earlier studies suggested that TAMs participate in antitumor responses; however, recent evidences indicate that they support tumor progression by blocking the antitumor immunity and by secreting factors that promote angiogenesis and re-activation of the epithelial-to-mesenchymal transition, which enhance metastasis formation [39]. In BC, TAMs are the most abundant inflammatory cells and are typically M2-polarized with suppressive capacity [39] that stems from their enzymatic activities and production of anti-inflammatory cytokines, such as TGFβ [46]. In our TNBC mouse model, M2-polarized macrophages and TGFβ expression were decreased after F1 treatment. High TAM levels have been associated with poorer BC outcomes [37]. Therefore, several strategies are currently under investigation, such as the suppression of TAM recruitment, their depletion, or the switch from the protumor M2 to the antitumor M1 phenotype in patients with TNBC [47]. Our findings showing reduced macrophage infiltration and decreased M2-like macrophages in response to F1 treatment are in line with the ongoing therapeutic strategies.

MDSCs are immature myeloid cells that promote the immunosuppressive tumor microenvironment through multiple mechanisms, including expression of immunosuppressive cytokines, such as TGFβ [48]. Treatment with F1 limited MDSC infiltration in MDA-MB-231 xenografts, and downregulated TGFβ expression. MDSC expansion in tumors is a major mechanism of tumor immune escape [38], and MDSC is considered as a potential cancer biomarker and therapeutic target [48]. Accordingly, disruption of MDSC function can improve the antitumor immune responses and impair tumor growth in mice [38]. Collectively, our data indicate that F1 treatment can specifically modulate myeloid cells in the tumor microenvironment, leading to a less immunosuppressive tumor microenvironment. As TAMs and MDSCs can suppress NK responses, it is possible that their depletion upon F1 therapy favors NK activation and cytotoxic activity, particularly through TGFβ downregulation. Consequently, we suggest that treatment with the anti-cath-D F1 antibody could normalize the tumor microenvironment, by boosting the host antitumor immune response, as illustrated here by NK cell activation. Our data show that F1 Fc effector function has an impact on its antitumor effect through NK cell dependent-pathways. However, we do not know how F1 limits TAM and MDSC infiltration in tumors. As in vitro cath-D can cleave chemokines that are implicated in the immune responses and the recruitment of myeloid cells and lymphocytes [16, 17], the Fab part of F1 could affect TAM and MDSC recruitment by modulating chemokine homeostasis.

In this study, to generate clinically relevant TNBC mouse models (MDA-MB-231 cell xenografts and PDXs), we used immunodeficient Foxn1^nu^ nude mice, instead of severely immunocompromised mice strains with little or no endogenous immune system, such as NOD/SCID, BAL-B/c-RAG2^null^, or their derivatives [49]. The use of the more clinically relevant Foxn1^nu^ mouse model that harbors NK and myeloid cell populations allowed the discovery of F1 immunomodulatory activity. However, this model did not permit to assess F1 effect on lymphocyte populations. Thus, our future studies will focus on humanized mouse models.

## Conclusion

This study demonstrates for the first time that cath-D is a tumor-specific extracellular target in TNBC and its suitability for antibody-based therapy. Furthermore, our preclinical proof-of-concept study validates the feasibility and efficacy of an immunomodulatory antibody-based strategy against cath-D to treat patients with TNBC.

## Additional files

Additional file 1: **Table S1.** Nucleotide sequences of the primers used for qPCR experiments. (PDF 163 kb)

Additional file 2: **Figure S1.** Cath-D expression in different BC subtypes. Total cath-D expression was determined in 159 whole cytosols from primary BC biopsies (HR^+^/HER2^+^ (*n* = 38); HR^-^/HER2^+^ (*n* = 38); HR^+^/HER2^-^ (*n* = 42); HR^-^/HER^-^ (*n* = 41)) by sandwich ELISA with the immobilized anti-human cath-D D7E3 antibody and the anti-human cath-D M1G8 antibody coupled to HRP. HR= ER + PR. Mean ± SEM. (PPTX 61 kb)

Additional file 3: **Figure S2.** Schematic overview of the biotinylation method used for the identification of accessible proteins in TNBC samples.The technique involves the biotinylation of TNBC biopsies by immersion in the chemically modified biotin solution. Proteins are then solubilized and biotinylated proteins are captured on the streptavidin material. After their enrichment, these proteins can be analyzed using HPLC chromatographic separation, MS analysis, selection of a particular mass (peptide), and fragmentation (MS/MS). MS/MS yields a pattern that delivers the sequence of the peptides and contributes to protein identification. The putative biomarkers discovered by this method require subsequent validation, for instance by immunohistochemistry. (PPTX 42 kb)

Additional file 4: **Figure S3.** Representative image of cath-D expression in TNBC biopsies. Cath-D expression was monitored by IHC using monoclonal anti-human cath-D (C-5; sc-377127) antibody in TMA. Staining is prominent in breast cancer cells and is also detected in the tumor stroma. Scale bar, 100 μm. (PPTX 2200 kb)

Additional file 5: **Figure S4.** Generation of anti-cath-D human scFv fragments by phage display. (A) Enrichment of anti-cath-D polyclonal scFv fragments by phage display. ScFv phages specific for human mature 34+14-kDa cath-D were selected and enriched in four biopanning rounds, and analyzed by ELISA using a HRP-labeled anti-M13 antibody. BSA, negative antigen. (B) Selection of anti-cath-D monoclonal scFv fragments by ELISA. ELISA performed using bacterial culture supernatants of the best scFv clones (5 out of 400 screened clones) and recombinant human mature 34+14-kDa cath-D and 52-kDa pro-cath-D. Binding of the scFv clones to cath-D was detected with a HRP-labeled anti-Myc antibody. BSA, negative antigen; IR, irrelevant scFv from the screen. (C) Purification of the anti-human cath-D scFv fragments. His-tagged anti-cath-D scFv fragments were purified using TALON resin, resolved by 12% SDS-PAGE and stained with Coomassie blue. (D) Binding of purified anti-cath-D monoclonal scFv antibodies to human cath-D from MDA-MB-231 cells. Binding of purified anti-cath-D scFv antibodies to secreted pro-cath-D and cellular cath-D from MDA-MB-231 cells was assayed by ELISA using an anti-His HRP-conjugated antibody (left panel). BSA, negative antigen; IR, irrelevant scFv; *n* = 3 Right panel, a whole cell lysate (10 μg) and conditioned medium (80 μl) from MDA-MB-231 cells were analyzed by 12% SDS-PAGE and immunoblotting using a polyclonal anti-mouse cath-D (sc-6486) antibody that cross-reacts with human cath-D (52-, 48- and 34-kDa isoforms). *Mr*, relative molecular mass (kDa). (E) Anti-human cath-D monoclonal scFv antibodies cross-react with mouse cath-D. Binding of anti-cath-D scFv antibodies to cath-D from mouse embryonic fibroblasts (MEFs) was monitored by ELISA using an anti His HRP-conjugated antibody (left panel). BSA, negative antigen; *n* = 3. Right panel, whole mouse embryonic fibroblast lysate (25 μg) was analyzed by 12% SDS-PAGE and immunoblotting using a polyclonal anti-mouse cath-D (sc-6486) antibody against the mouse cellular cath-D 48- and 34-kDa isoforms. *Mr*, relative molecular mass (kDa). (PPTX 433 kb)

Additional file 6: **Figure S5.** Selectivity of F1 and E2 towards pro-cath-D. Indirect ELISA was performed with recombinant human pro-cath-D, pro-cathepsin E, pepsinogen A and pepsinogen C. BSA, negative antigen. RTX, rituximab (negative control antibody). (PPTX 59 kb)

Additional file 7: **Figure S6.** Biodistribution analysis of anti-cath-D F1 and E2 human antibodies. Nude mice bearing subcutaneous MDA-MB-231 tumor cell xenografts received one injection of ^177^Lu- F1 (upper panel) or ^177^Lu- E2 (lower panel). The percentage of injected activity per gram of tissue (%IA/g tissue; mean ± SD) was determined in healthy organs and tumors at 24, 48, 72 and 96 hours post-injection. (PPTX 753 kb)

Additional file 8: **Figure S7.** Effect of the F1 and E2 antibodies on mouse weight. Mean mouse weight during F1 and E2 treatment. Mice from Fig. 4a were weighted during F1, E2 or rituximab (CRTL) treatment (15mg/kg three times per week for 28 days); *n* = 9 per group. (PPTX 64 kb)

Additional file 9: **Figure S8.** Effect of F1 and E2 on tumor cell proliferation, apoptosis, and angiogenesis in MDA-MB-231 tumor cell xenografts. (A) Ki67 immunostaining. Representative images in tumors from CTRL- (rituximab), F1- and E2-treated mice. Scale bars, 100 μm. (B) Quantification of Ki67. Percentage (mean ± SEM) of Ki67-positive cells relative to total cell number (*n* = 9 for rituximab (CTRL); *n* = 9 for F1; *n* = 9 for E2). (C) Activated caspase 3 immunostaining. Representative images in tumors from CTRL- (rituximab), F1- and E2-treated mice. Scale bars, 100 μm. (D) Quantification of activated caspase 3. Percentage (mean ± SEM) of activated caspase 3-positive pixels relative to total pixels (*n* = 9 for rituximab (CTRL); *n* = 9 for F1; *n* = 9 for E2). (E) CD31 immunostaining. Representative images in tumors from CTRL- (rituximab), F1- and E2-treated mice. Scale bars, 100 μm. (F) Quantification of CD31. Percentage (mean ± SEM) of CD31 cells/field (*n* = 9 for rituximab (CTRL); *n* = 9 for F1; *n* = 9 for E2). (PPTX 1660 kb)

Additional file 10: **Figure S9.** Binding of F1Fc to pro-cath-D secreted from MDA-MB-231 cells. Sandwich ELISA in which pro-cath-D from conditioned medium of MDA-MB-231 cells was added to wells pre-coated with the anti-pro-cath-D M2E8 mouse monoclonal antibody in the presence of F1Fc (1μg/ml) or F1 (1μg/ml). Binding of F1Fc and F1 to pro-cath-D was revealed with an anti-human Fc antibody conjugated to HRP. RTX, rituximab (negative control antibody). (PPTX 56 kb)

## Abbreviations

ADCC: antibody-dependent cellular cytotoxicity
BC: breast cancer
cath-D: cathepsin D
ER: estrogen receptor
FACS: Fluorescence Activated Cell Sorting
Fc: fragment crystallizable
FCS: fetal calf serum
HBSS: Hank’s Balanced Salt Solution
HER-2: human epidermal growth factor receptor 2
HR: hazard ratio
HRP: horseradish peroxidase
IHC: immunohistochemistry
MDSCs: myeloid-derived suppressor cells
PDXs: patient-derived xenografts
PR: progesterone receptor
RT-qPCR: Quantitative reverse transcription PCR
scFv: single-chain variable fragment
SPECT/CT: Single Photon Emission Computed Tomography
TAMs: tumor-associated macrophages
TMA: Tissue Micro-Array
TNBC: Triple-negative breast cancer
