# Supplementary material for: Immunotherapy of triple-negative breast cancer with cathepsin D-targeting antibodies
Source: J Immunother Cancer. 2019 Feb 4;7:29. doi: 10.1186/s40425-019-0498-z (PMC6360707; doi:10.1186/s40425-019-0498-z)
Supplement: Supplementary file 1 — Table S1. Nucleotide sequences of the primers used for qPCR experiments. (PDF 163 kb) [file 40425_2019_498_MOESM1_ESM.pdf]

Name	Sequence
IL-15	forward 5'AAAGGCATTCCAGGACA3'<br>reverse 5'CCAACCCCAGCTAACAG3'
GZMB	forward 5'CCTCCTGCTACTGCTGAC 3'<br>reverse 5'GTCAGCACAAAGTCCTCTC 3'
PRF1	forward 5'GAGAAGACCTATCAGGACCA3'<br>reverse 5'AGCCTGTGGTAAGCATG3'
IFNγ	forward 5'ACAATGAACGCTACACACTGCAT3'<br>reverse 5'TGGCAGTAACAGCCAGAAACA3'
CD206	forward 5'CTCTGTTCAGCTATT3'<br>reverse 5'CGGAATTTCTGGGAT 3'
F4/80	forward 5'CTTTGGCTATGGGCT3'<br>reverse 5'GCAAGGAGGACAGAG3'
TGFβ	forward 5'GAGGTCACCCGCGTGCTA3'<br>reverse 5' TGTGTGAGATGTCTTTGGTTTTCTC3'
RPS9	forward 5'CGGCCCGCGAGCTGT3'<br>reverse 5'CTGCTTGCGGACCCT3'

Supplemental Table 1. Nucleotide sequences of the primers used for qPCR experiments.
