# Supplementary figures and images for: Immunotherapy of triple-negative breast cancer with cathepsin D-targeting antibodies
Source: J Immunother Cancer. 2019 Feb 4;7:29. doi: 10.1186/s40425-019-0498-z (PMC6360707; doi:10.1186/s40425-019-0498-z)

## Slide 1
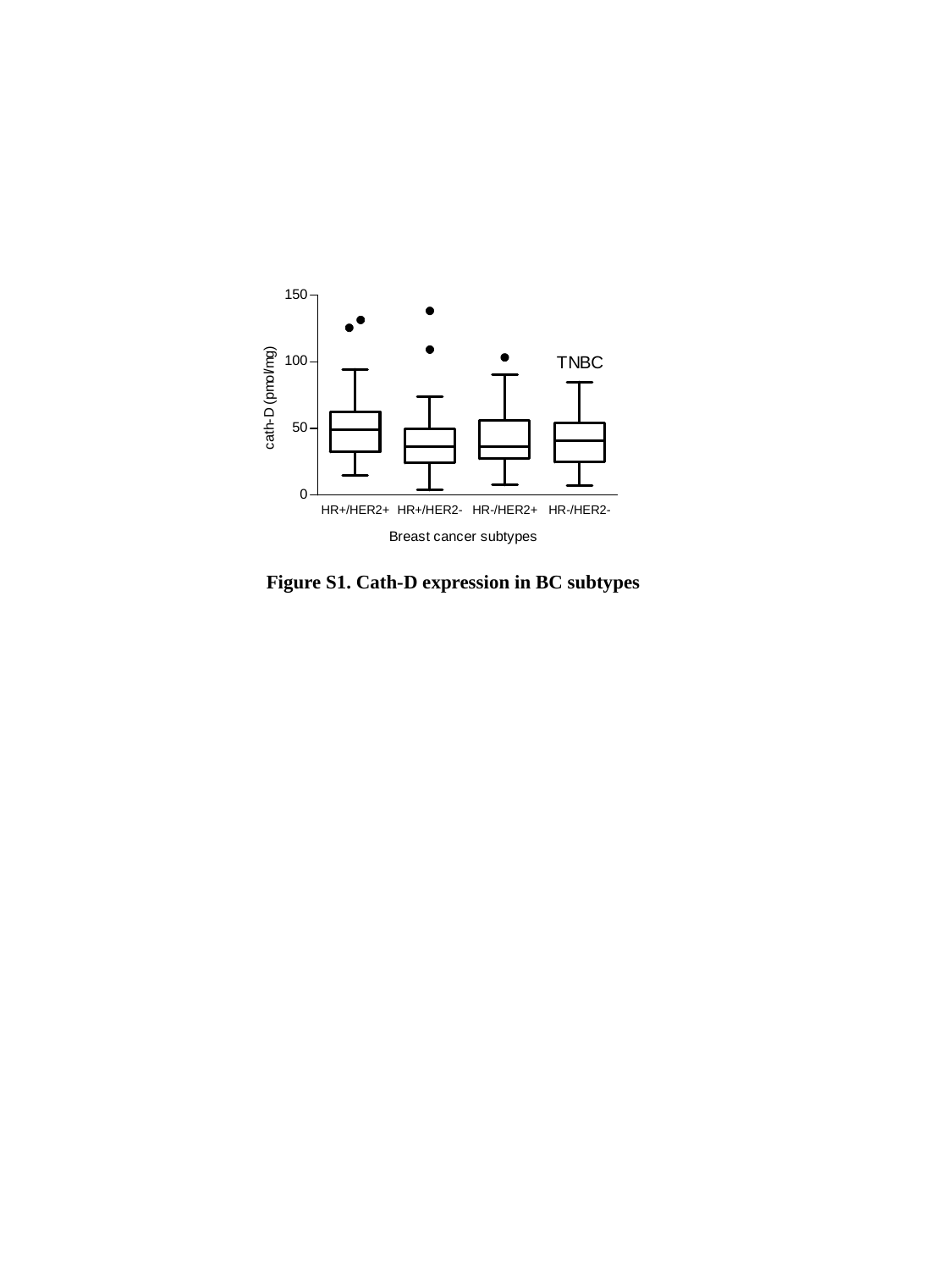

Figure S1. Cath-D expression in BC subtypes

Supplement: Supplementary file 2 — Figure S1. Cath-D expression in different BC subtypes. Total cath-D expression was determined in 159 whole cytosols from primary BC biopsies (HR+/HER2+ (n = 38); HR-/HER2+ (n = 38); HR+/HER2- (n = 42); HR-/HER- (n = 41)) by sandwich ELISA with the immobilized anti-human cath-D D7E3 antibody and the anti-human cath-D M1G8 antibody coupled to HRP. HR= ER + PR. Mean ± SEM. (PPTX 61 kb) [file 40425_2019_498_MOESM2_ESM.pptx]

## Slide 1
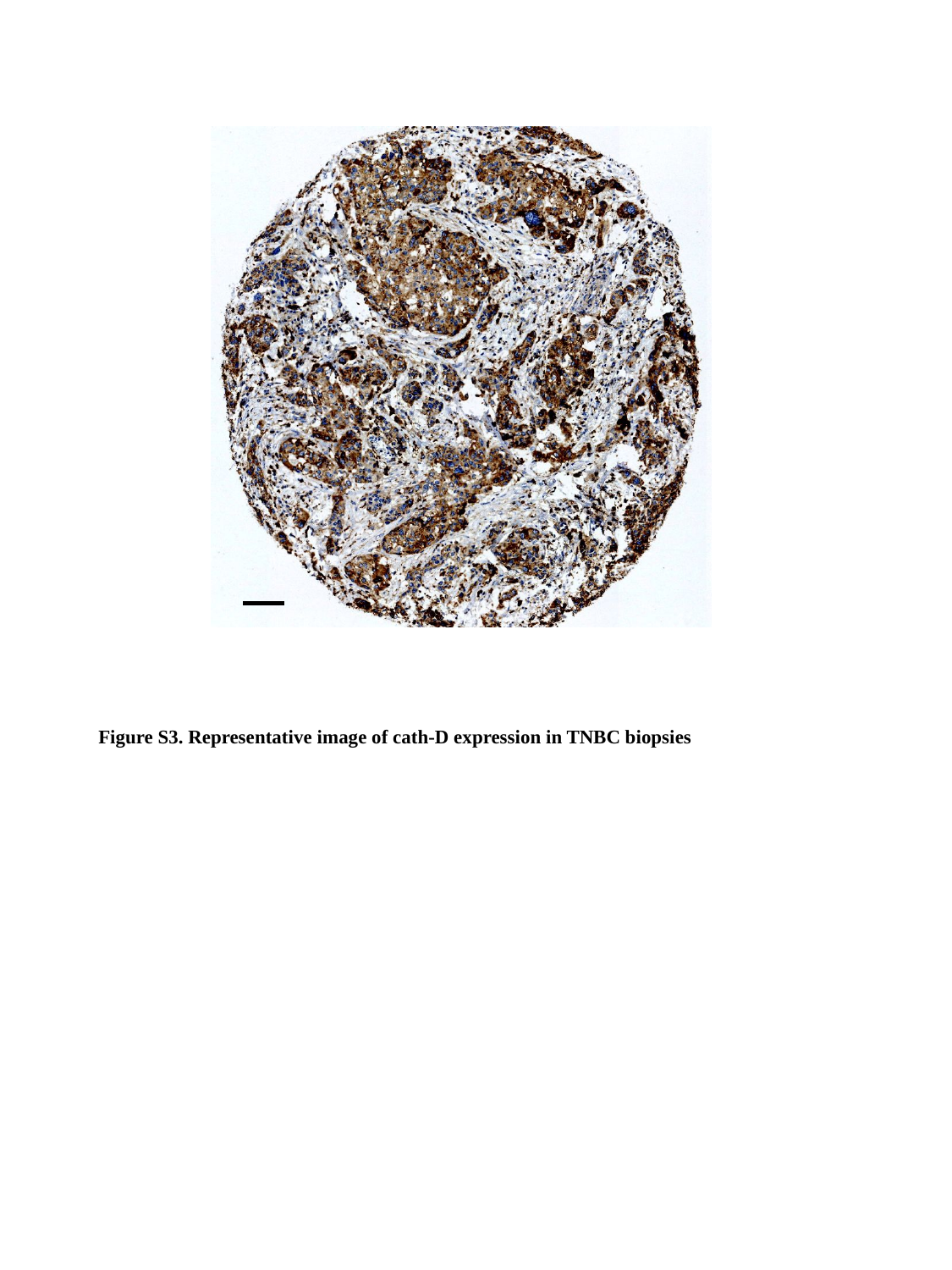

Stroma
T
Figure S3. Representative image of cath-D expression in TNBC biopsies

Supplement: Supplementary file 4 — Figure S3. Representative image of cath-D expression in TNBC biopsies. Cath-D expression was monitored by IHC using monoclonal anti-human cath-D (C-5; sc-377127) antibody in TMA. Staining is prominent in breast cancer cells and is also detected in the tumor stroma. Scale bar, 100 μm. (PPTX 2200 kb) [file 40425_2019_498_MOESM4_ESM.pptx]

## Slide 1
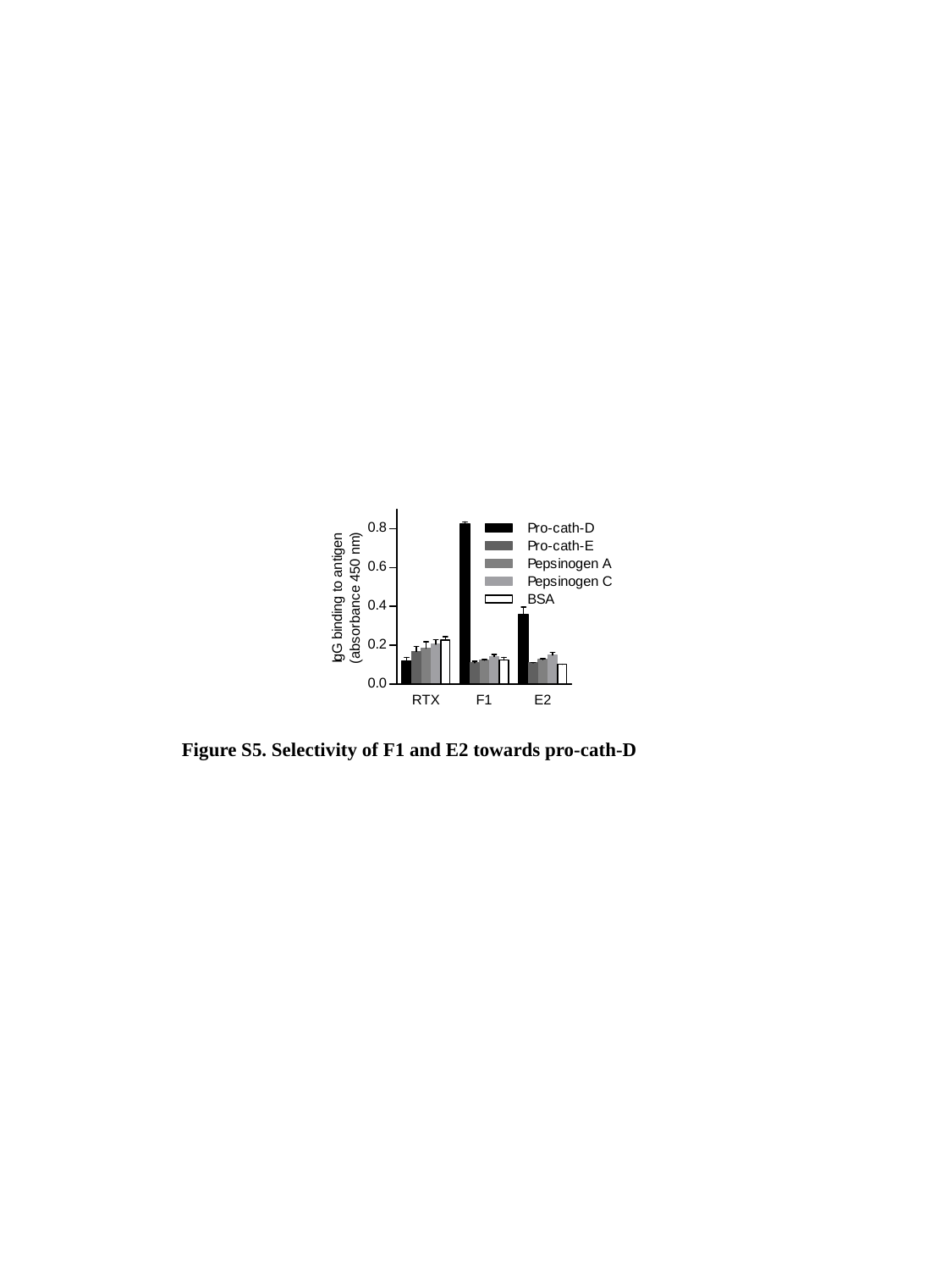

Figure S5. Selectivity of F1 and E2 towards pro-cath-D

Supplement: Supplementary file 6 — Figure S5. Selectivity of F1 and E2 towards pro-cath-D. Indirect ELISA was performed with recombinant human pro-cath-D, pro-cathepsin E, pepsinogen A and pepsinogen C. BSA, negative antigen. RTX, rituximab (negative control antibody). (PPTX 59 kb) [file 40425_2019_498_MOESM6_ESM.pptx]

## Slide 1
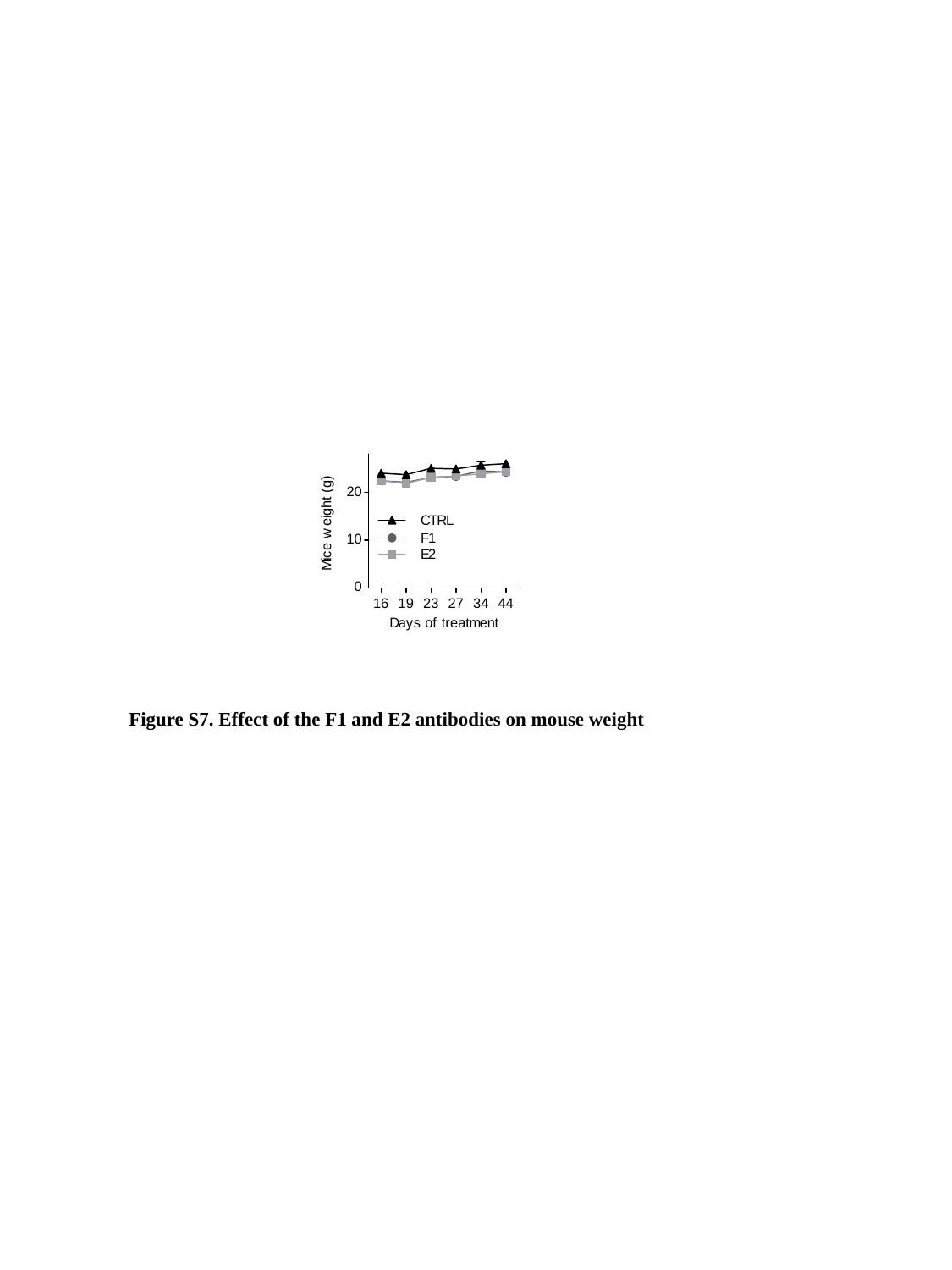

Figure S7. Effect of the F1 and E2 antibodies on mouse weight

Supplement: Supplementary file 8 — Figure S7. Effect of the F1 and E2 antibodies on mouse weight. Mean mouse weight during F1 and E2 treatment. Mice from Fig. 4a were weighted during F1, E2 or rituximab (CRTL) treatment (15mg/kg three times per week for 28 days); n = 9 per group. (PPTX 64 kb) [file 40425_2019_498_MOESM8_ESM.pptx]

## Slide 1
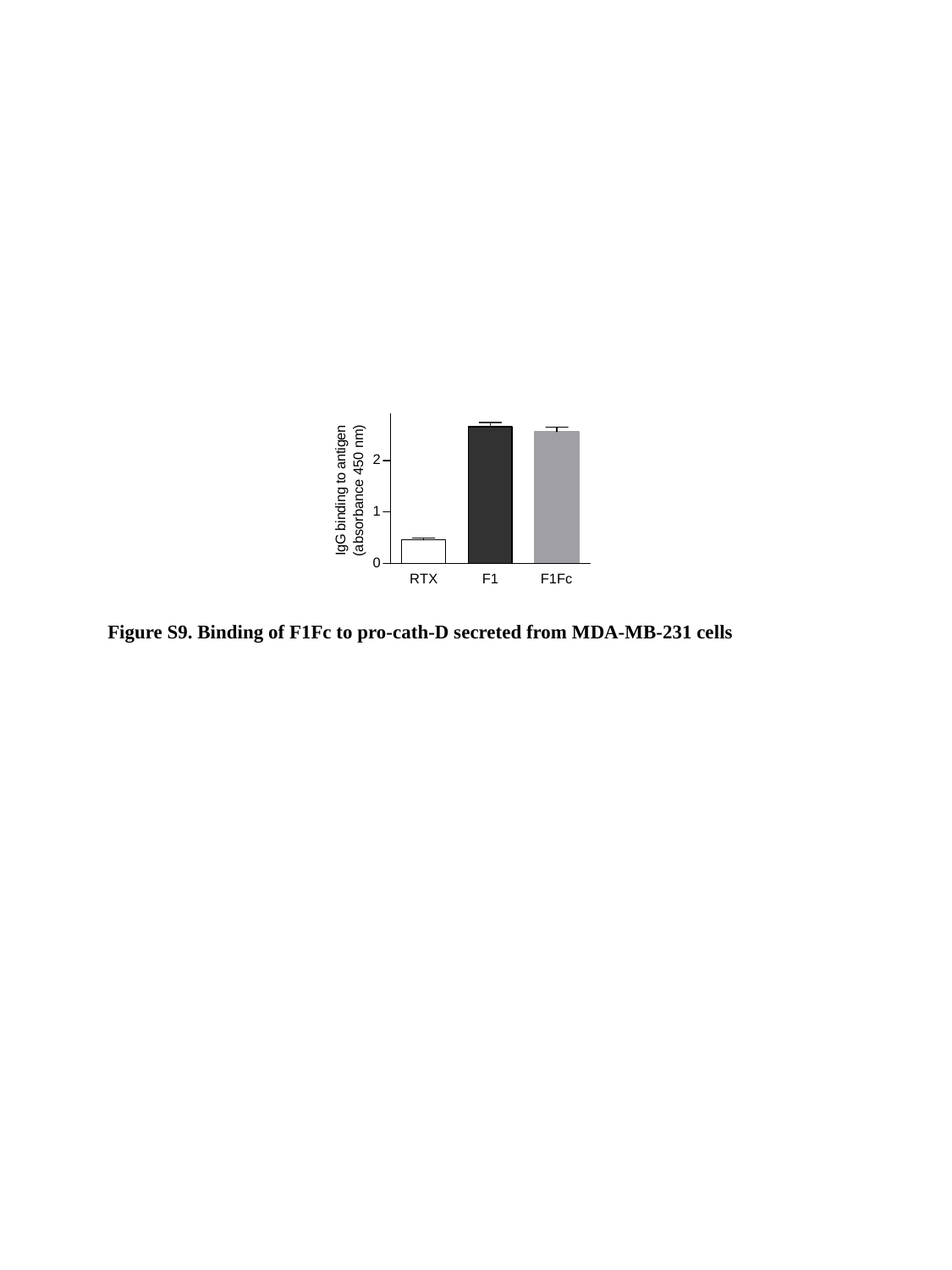

Figure S9. Binding of F1Fc to pro-cath-D secreted from MDA-MB-231 cells

Supplement: Supplementary file 10 — Figure S9. Binding of F1Fc to pro-cath-D secreted from MDA-MB-231 cells. Sandwich ELISA in which pro-cath-D from conditioned medium of MDA-MB-231 cells was added to wells pre-coated with the anti-pro-cath-D M2E8 mouse monoclonal antibody in the presence of F1Fc (1μg/ml) or F1 (1μg/ml). Binding of F1Fc and F1 to pro-cath-D was revealed with an anti-human Fc antibody conjugated to HRP. RTX, rituximab (negative control antibody). (PPTX 56 kb) [file 40425_2019_498_MOESM10_ESM.pptx]
