# Supplementary material for: Immunotherapy of triple-negative breast cancer with cathepsin D-targeting antibodies
Source: J Immunother Cancer. 2019 Feb 4;7:29. doi: 10.1186/s40425-019-0498-z (PMC6360707; doi:10.1186/s40425-019-0498-z)
Supplement: Supplementary file 3 — Figure S2. Schematic overview of the biotinylation method used for the identification of accessible proteins in TNBC samples.The technique involves the biotinylation of TNBC biopsies by immersion in the chemically modified biotin solution. Proteins are then solubilized and biotinylated proteins are captured on the streptavidin material. After their enrichment, these proteins can be analyzed using HPLC chromatographic separation, MS analysis, selection of a particular mass (peptide), and fragmentation (MS/MS). MS/MS yields a pattern that delivers the sequence of the peptides and contributes to protein identification. The putative biomarkers discovered by this method require subsequent validation, for instance by immunohistochemistry. (PPTX 42 kb) [file 40425_2019_498_MOESM3_ESM.pptx]

## Slide 1
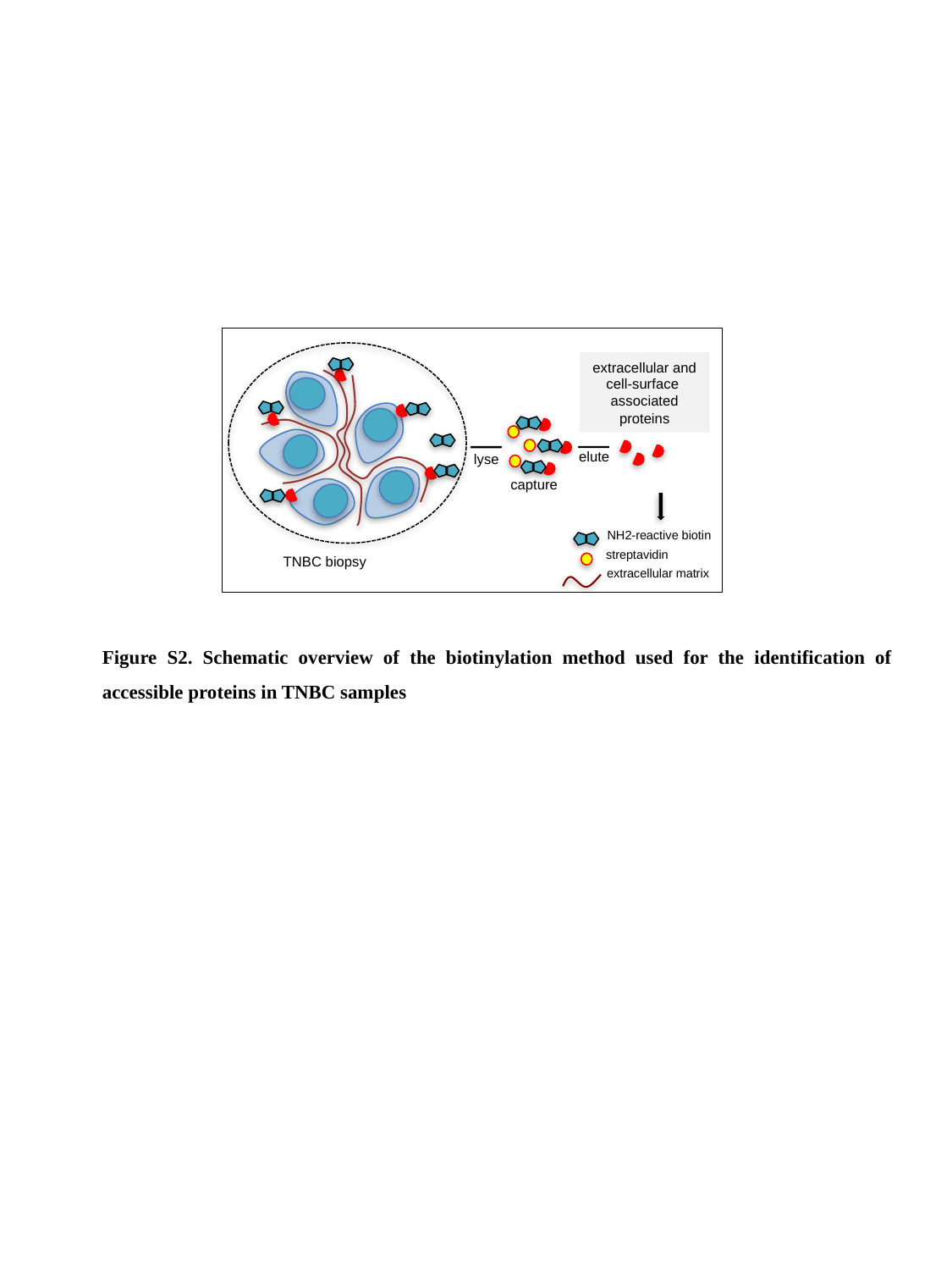

extracellular and
cell-surface
associated proteins
elute
lyse
capture
NH2-reactive biotin
streptavidin
TNBC biopsy
extracellular matrix
Figure S2. Schematic overview of the biotinylation method used for the identification of accessible proteins in TNBC samples
