# Supplementary material for: Immunotherapy of triple-negative breast cancer with cathepsin D-targeting antibodies
Source: J Immunother Cancer. 2019 Feb 4;7:29. doi: 10.1186/s40425-019-0498-z (PMC6360707; doi:10.1186/s40425-019-0498-z)
Supplement: Supplementary file 5 — Figure S4. Generation of anti-cath-D human scFv fragments by phage display. (A) Enrichment of anti-cath-D polyclonal scFv fragments by phage display. ScFv phages specific for human mature 34+14-kDa cath-D were selected and enriched in four biopanning rounds, and analyzed by ELISA using a HRP-labeled anti-M13 antibody. BSA, negative antigen. (B) Selection of anti-cath-D monoclonal scFv fragments by ELISA. ELISA performed using bacterial culture supernatants of the best scFv clones (5 out of 400 screened clones) and recombinant human mature 34+14-kDa cath-D and 52-kDa pro-cath-D. Binding of the scFv clones to cath-D was detected with a HRP-labeled anti-Myc antibody. BSA, negative antigen; IR, irrelevant scFv from the screen. (C) Purification of the anti-human cath-D scFv fragments. His-tagged anti-cath-D scFv fragments were purified using TALON resin, resolved by 12% SDS-PAGE and stained with Coomassie blue. (D) Binding of purified anti-cath-D monoclonal scFv antibodies to human cath-D from MDA-MB-231 cells. Binding of purified anti-cath-D scFv antibodies to secreted pro-cath-D and cellular cath-D from MDA-MB-231 cells was assayed by ELISA using an anti-His HRP-conjugated antibody (left panel). BSA, negative antigen; IR, irrelevant scFv; n = 3 Right panel, a whole cell lysate (10 μg) and conditioned medium (80 μl) from MDA-MB-231 cells were analyzed by 12% SDS-PAGE and immunoblotting using a polyclonal anti-mouse cath-D (sc-6486) antibody that cross-reacts with human cath-D (52-, 48- and 34-kDa isoforms). Mr, relative molecular mass (kDa). (E) Anti-human cath-D monoclonal scFv antibodies cross-react with mouse cath-D. Binding of anti-cath-D scFv antibodies to cath-D from mouse embryonic fibroblasts (MEFs) was monitored by ELISA using an anti His HRP-conjugated antibody (left panel). BSA, negative antigen; n = 3. Right panel, whole mouse embryonic fibroblast lysate (25 μg) was analyzed by 12% SDS-PAGE and immunoblotting using a polyclonal anti-mouse cath-D (sc-6486) an [file 40425_2019_498_MOESM5_ESM.pptx]

## Slide 1
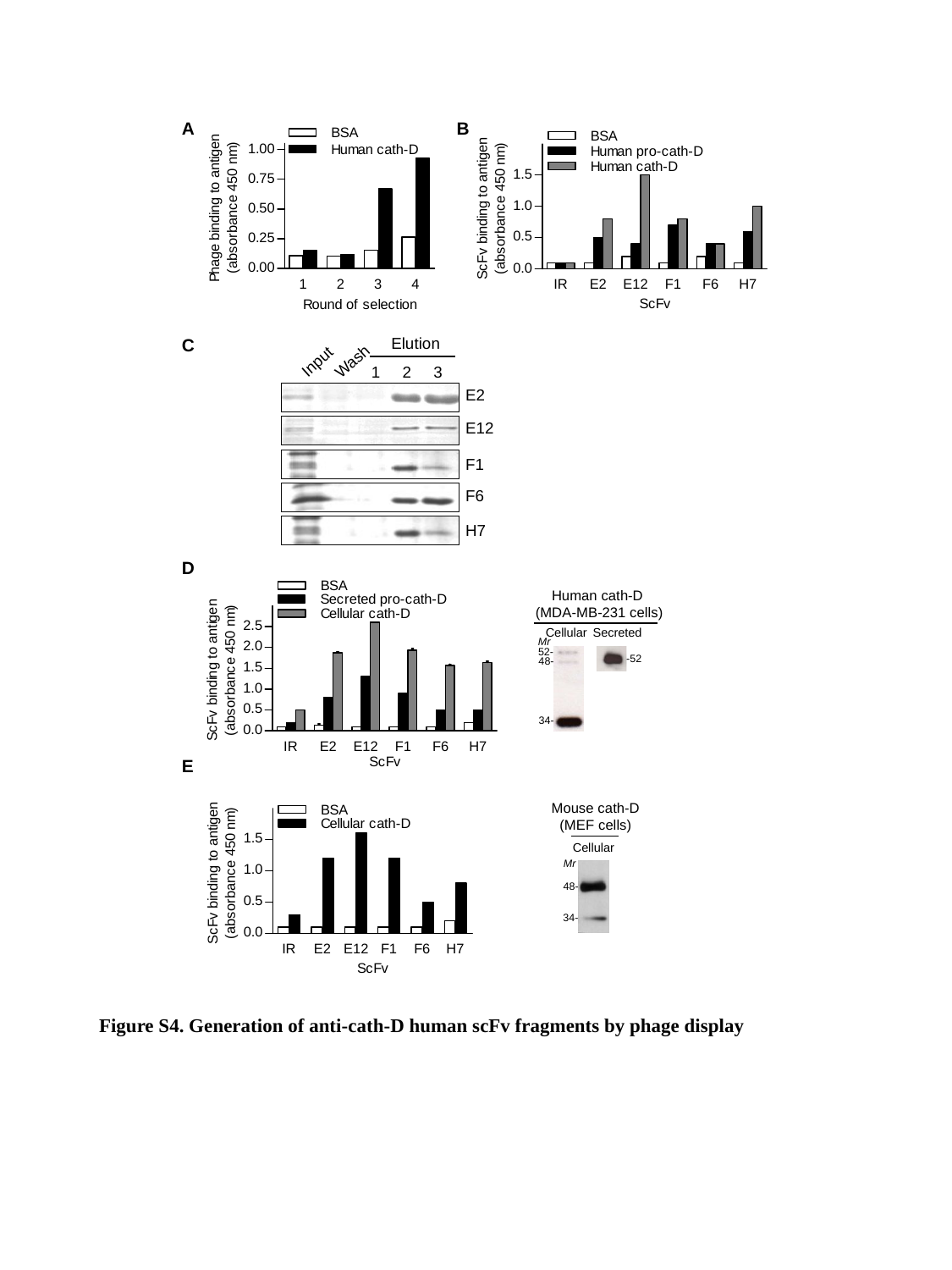

A
B
Elution
Input
Wash
1 2 3
E2
E12
F1
F6
H7
C
D
Human cath-D
(MDA-MB-231 cells)
Cellular
Secreted
Mr
52-
-52
48-
34-
E
Mouse cath-D
(MEF cells)
Cellular
48-
34-
Mr
Figure S4. Generation of anti-cath-D human scFv fragments by phage display
