# Supplementary material for: Immunotherapy of triple-negative breast cancer with cathepsin D-targeting antibodies
Source: J Immunother Cancer. 2019 Feb 4;7:29. doi: 10.1186/s40425-019-0498-z (PMC6360707; doi:10.1186/s40425-019-0498-z)
Supplement: Supplementary file 7 — Figure S6. Biodistribution analysis of anti-cath-D F1 and E2 human antibodies. Nude mice bearing subcutaneous MDA-MB-231 tumor cell xenografts received one injection of 177Lu- F1 (upper panel) or 177Lu- E2 (lower panel). The percentage of injected activity per gram of tissue (%IA/g tissue; mean ± SD) was determined in healthy organs and tumors at 24, 48, 72 and 96 hours post-injection. (PPTX 753 kb) [file 40425_2019_498_MOESM7_ESM.pptx]

## Slide 1
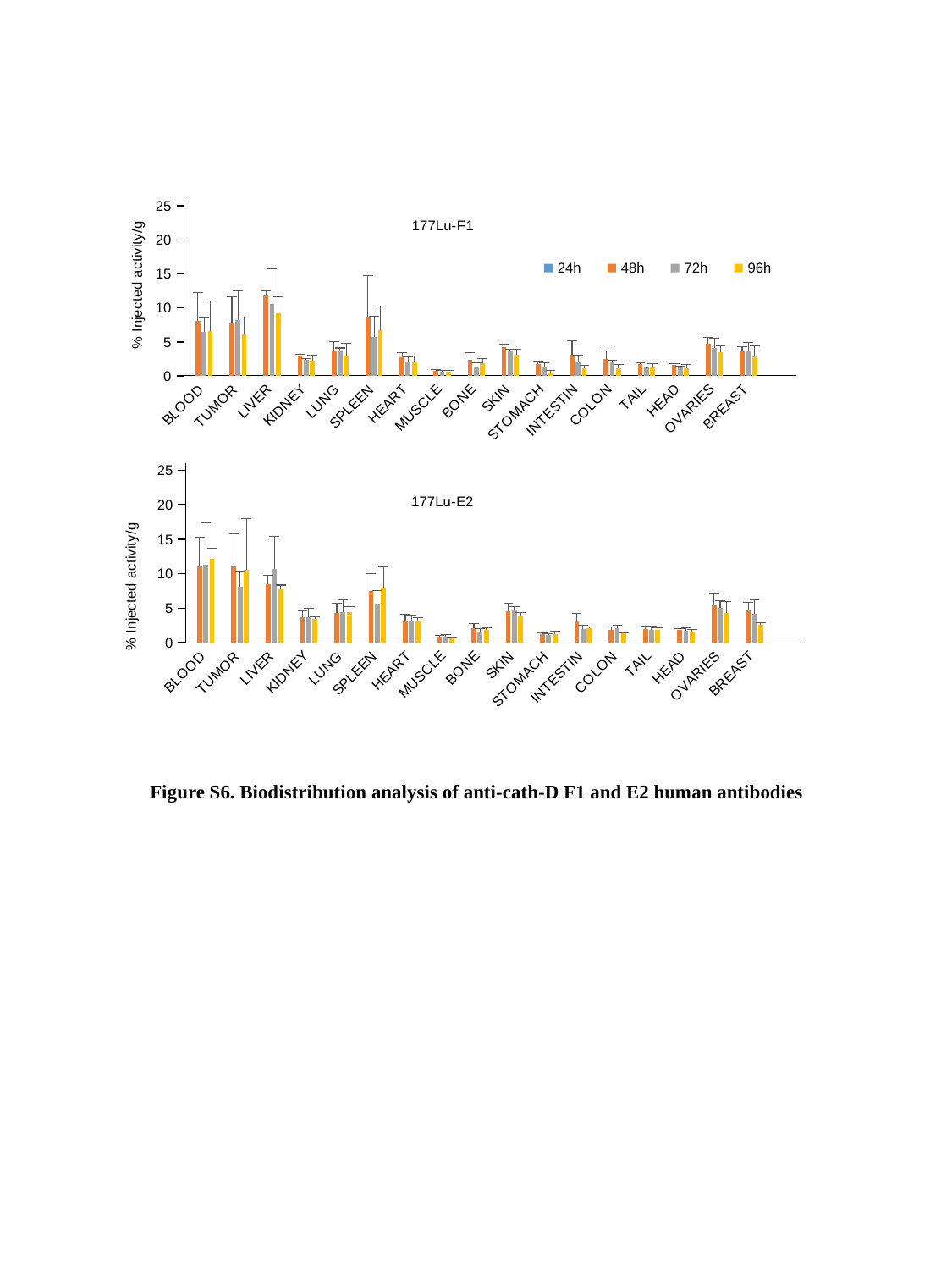

### Chart: 177Lu-F1
| Category | 24h | 48h | 72h | 96h |
|---|---|---|---|---|
| BLOOD | 12.429988795232223 | 8.03743781051958 | 6.403676198488081 | 6.530304043852924 |
| TUMOR | 5.206387586549669 | 7.889727749301441 | 8.190325308586651 | 6.052894079959761 |
| LIVER | 19.508138317092197 | 11.856185225133673 | 10.526581166481035 | 9.16986692312448 |
| KIDNEY | 3.991667821286959 | 3.0369225462809752 | 2.3077779349176106 | 2.2267752008882202 |
| LUNG | 5.172537204228518 | 3.738628146498491 | 3.594356815049207 | 2.9209981705489882 |
| SPLEEN | 6.612570313048618 | 8.529452990293791 | 5.706625303464938 | 6.661704410685251 |
| HEART | 3.608474839185721 | 2.7462250859080397 | 2.0689307694601045 | 1.9432075379402172 |
| MUSCLE | 1.0282931346869282 | 0.7339109846169343 | 0.6237619698926307 | 0.6367221243933237 |
| BONE | 2.0713699584329355 | 2.30602784421648 | 1.3978616478690873 | 1.8828991962909227 |
| SKIN | 4.864155000159471 | 4.188641326061037 | 3.6957429613525177 | 3.1556994894636303 |
| STOMACH | 1.2601556029608822 | 1.6860469557778184 | 1.2610994674364433 | 0.5350849008027883 |
| INTESTIN | 3.131840357391937 | 3.074743092251455 | 2.0181601269734304 | 1.1488646076948716 |
| COLON | 5.663880890248713 | 2.5308943750260697 | 2.075454979166466 | 1.103067911038102 |
| TAIL | 1.6107728843155291 | 1.7184393600419332 | 1.2234904517511613 | 1.26409687161548 |
| HEAD | 1.9541848472947434 | 1.6071899786255437 | 1.274753960583066 | 1.1595837044127508 |
| OVARIES | 4.781029189863297 | 4.691538957591517 | 4.050669090003573 | 3.4551415070953135 |
| BREAST | 5.392141528794042 | 3.6430510370774956 | 3.59796074457784 | 2.8174565969486123 |
### Chart: 177Lu-E2
| Category | 24h | 48h | 72h | 96h |
|---|---|---|---|---|
| BLOOD | 17.224814131235515 | 11.050728730380492 | 11.330114181335242 | 12.19556428252542 |
| TUMOR | 6.585290782662266 | 11.087510516683672 | 8.097832599806182 | 10.545180458538757 |
| LIVER | 15.02104382761975 | 8.535572667230928 | 10.669510135676067 | 7.727819664900953 |
| KIDNEY | 4.434060493266202 | 3.6678554403561288 | 3.660667246672949 | 3.4304842333903385 |
| LUNG | 6.488199783719391 | 4.341368310769015 | 4.485530109766925 | 4.414596813297385 |
| SPLEEN | 5.367666998023309 | 7.477089492239655 | 5.626525718658813 | 7.943197552541505 |
| HEART | 4.470256338226623 | 3.0794810660629466 | 3.026971111357323 | 3.0723112864069493 |
| MUSCLE | 1.2916402545165317 | 0.9261289863652349 | 0.8181324447129827 | 0.7537797945428197 |
| BONE | 2.053199858552304 | 2.1557591010503745 | 1.6458602453840558 | 1.8594263466787195 |
| SKIN | 5.033418642955689 | 4.6075440758135455 | 4.803005735242155 | 3.7607074411098993 |
| STOMACH | 1.7810543840935067 | 1.2580889184103565 | 1.0846414763142964 | 1.2306186874488443 |
| INTESTIN | 3.9365709031725182 | 3.125708559084415 | 1.943593740833517 | 2.13565176325134 |
| COLON | 4.7247348146096675 | 1.908543715480449 | 2.098256848497223 | 1.4104168622742552 |
| TAIL | 2.0297582383109094 | 1.9627964586796085 | 1.8870723762899342 | 1.798709564278403 |
| HEAD | 2.29909726489838 | 1.8235565730806649 | 1.7473296680173034 | 1.6542348305316594 |
| OVARIES | 4.527444740912872 | 5.416870663281747 | 5.0400260791985225 | 4.251592524520486 |
| BREAST | 3.2808786431704937 | 4.686330377456255 | 4.15959874161384 | 2.5391371947977532 |
Figure S6. Biodistribution analysis of anti-cath-D F1 and E2 human antibodies
