# Supplementary material for: Immunotherapy of triple-negative breast cancer with cathepsin D-targeting antibodies
Source: J Immunother Cancer. 2019 Feb 4;7:29. doi: 10.1186/s40425-019-0498-z (PMC6360707; doi:10.1186/s40425-019-0498-z)
Supplement: Supplementary file 9 — Figure S8. Effect of F1 and E2 on tumor cell proliferation, apoptosis, and angiogenesis in MDA-MB-231 tumor cell xenografts. (A) Ki67 immunostaining. Representative images in tumors from CTRL- (rituximab), F1- and E2-treated mice. Scale bars, 100 μm. (B) Quantification of Ki67. Percentage (mean ± SEM) of Ki67-positive cells relative to total cell number (n = 9 for rituximab (CTRL); n = 9 for F1; n = 9 for E2). (C) Activated caspase 3 immunostaining. Representative images in tumors from CTRL- (rituximab), F1- and E2-treated mice. Scale bars, 100 μm. (D) Quantification of activated caspase 3. Percentage (mean ± SEM) of activated caspase 3-positive pixels relative to total pixels (n = 9 for rituximab (CTRL); n = 9 for F1; n = 9 for E2). (E) CD31 immunostaining. Representative images in tumors from CTRL- (rituximab), F1- and E2-treated mice. Scale bars, 100 μm. (F) Quantification of CD31. Percentage (mean ± SEM) of CD31 cells/field (n = 9 for rituximab (CTRL); n = 9 for F1; n = 9 for E2). (PPTX 1660 kb) [file 40425_2019_498_MOESM9_ESM.pptx]

## Slide 1
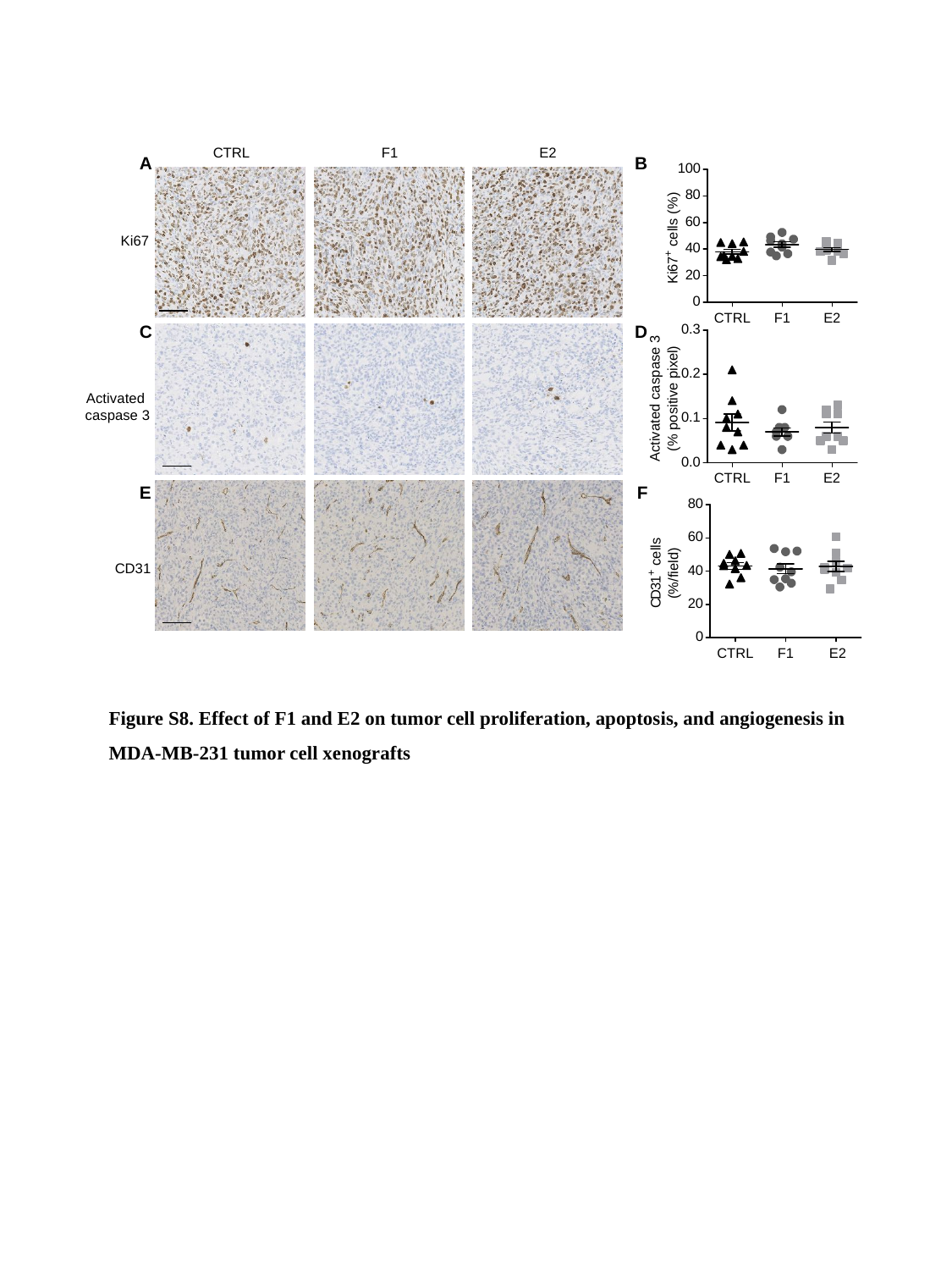

CTRL
F1
E2
A
B
Ki67
C
D
Activated
caspase 3
E
F
CD31
Figure S8. Effect of F1 and E2 on tumor cell proliferation, apoptosis, and angiogenesis in MDA-MB-231 tumor cell xenografts
